# Droplet Hi-C enables scalable, single-cell profiling of chromatin architecture in heterogeneous tissues

**DOI:** 10.1038/s41587-024-02447-1

**Published:** 2024-10-18

**Authors:** Lei Chang, Yang Xie, Brett Taylor, Zhaoning Wang, Jiachen Sun, Ethan J. Armand, Shreya Mishra, Jie Xu, Melodi Tastemel, Audrey Lie, Zane A. Gibbs, Hannah S. Indralingam, Tuyet M. Tan, Rafael Bejar, Clark C. Chen, Frank B. Furnari, Ming Hu, Bing Ren

**Affiliations:** 1 Department of Cellular and Molecular Medicine, University of California, San Diego, La Jolla, CA USA; 2 Biomedical Sciences Graduate Program, University of California, San Diego, La Jolla, CA USA; 3 Medical Scientist Training Program, University of California, San Diego, La Jolla, CA USA; 4 Department of Quantitative Health Sciences, Lerner Research Institute, Cleveland Clinic Foundation, Cleveland, OH USA; 5 Systems Biology and Bioinformatics PhD Program, Case Western Reserve University School of Medicine, Cleveland, OH USA; 6 Bioinformatics and Systems Biology Program, University of California, San Diego, La Jolla, CA USA; 7 Moores Cancer Center, UC San Diego, La Jolla, CA USA; 8 Department of Neurosurgery, University of Minnesota, Minneapolis, MN USA; 9 Department of Medicine, University of California, San Diego School of Medicine, La Jolla, CA USA; 10 Center for Epigenomics, Institute for Genomic Medicine, School of Medicine, University of California, San Diego, La Jolla, CA USA

**Keywords:** Next-generation sequencing, Predictive markers

## Abstract

Current methods for analyzing chromatin architecture are not readily scalable to heterogeneous tissues. Here we introduce Droplet Hi-C, which uses a commercial microfluidic device for high-throughput, single-cell chromatin conformation profiling in droplets. Using Droplet Hi-C, we mapped the chromatin architecture of the mouse cortex and analyzed gene regulatory programs in major cortical cell types. In addition, we used this technique to detect copy number variations, structural variations and extrachromosomal DNA in human glioblastoma, colorectal and blood cancer cells, revealing clonal dynamics and other oncogenic events during treatment. We refined the technique to allow joint profiling of chromatin architecture and transcriptome in single cells, facilitating exploration of the links between chromatin architecture and gene expression in both normal tissues and tumors. Thus, Droplet Hi-C both addresses critical gaps in chromatin analysis of heterogeneous tissues and enhances understanding of gene regulation.

## Abstract (web summary)

Chromatin organization is measured in single cells using droplet microfluidics.

## Main

Chromatin organization plays a crucial role in both embryonic development^1,2^ and the progression of various diseases^3,4^. During development, the chromatin structure influences which genes are turned on or off by the distal regulatory elements^5–8^. Abnormal chromatin organization can lead to aberrant gene expression or silencing, contributing to cancer development and metastasis^4^. Indeed, components of the nuclear machinery that regulates chromatin organization are among the most frequently mutated genes in human cancers^9–12^. Thus, a comprehensive analysis of the chromatin organization is crucial for understanding the gene regulatory programs involved in both normal development and disease pathogenesis.

Analyzing chromatin organization in primary tissues and tumor biopsies presents unique challenges owing to the complexity of chromatin dynamics and technical limitations. First, the biospecimens are often heterogeneous, containing multiple cell types. Current techniques such as bulk Hi-C could not resolve specific chromatin organization patterns relevant to particular cell types, especially in tumors in which cancer cells coexist with stromal and immune cells. Second, the data obtained from bulk chromatin organization assays are complex, and relating changes in chromatin organization to specific functional outcomes can be difficult. Addressing these challenges requires the development of more sensitive single-cell chromatin structure profiling assays and advanced computational tools for data analysis.

Great strides have been made in recent years in single-cell Hi-C technologies^1–4^. In general, single-cell Hi-C methods can be categorized into low-throughput microwell-based approaches and high-throughput combinatorial-indexing-based approaches. In the case of microwell-based single-cell Hi-C methods, cells or nuclei are individually dispensed into microwells, and library construction is carried out in each microwell in parallel, frequently with the help of automated liquid handlers. Notably, several single-cell Hi-C methods have been developed, such as single-cell Hi-C^13–15^, single-nucleus Hi-C^16^ and diploid chromatin conformation capture (Dip-C)^17^, and third-generation sequencing-based scNanoHi-C^18^. Recently, additional microwell-based single-cell Hi-C methods have emerged that combine Hi-C analysis with assays for one or more additional molecular modalities in a single cell. For example, Methyl-HiC^19^ and single-nucleus methyl-3C sequencing (sn-m3C-seq)^20^ capture chromatin interactions and DNA methylation patterns from the same cell. Hi-C and RNA sequencing employed simultaneously (HiRES)^21^ jointly performs Hi-C and RNA sequencing (RNA-seq) to explore the functional relationship between three-dimensional genome organization and transcriptome dynamics. In general, microwell-based techniques are limited in scale and difficult to adopt broadly owing to lengthy procedures and high cost. On the other hand, combinatorial-indexing-based single-cell Hi-C methods use combinatorial barcoding strategies to achieve high throughput and scalability. Previously, a single-cell combinatorial indexed Hi-C (sci-Hi-C)^22^ method allowed the generation of single-cell Hi-C libraries from a few thousand cells, albeit with limited genomic coverage in each cell. Building upon the same strategy, genome architecture and gene expression by sequencing (GAGE-seq)^23^ was developed to simultaneously profile chromatin interactions and gene expression in single cells, to achieve high throughput, multimodality and high coverage per cell. However, the lengthy and largely manual combinatorial indexing procedure still poses a challenge for its general adoption.

Cancer cells often show large-scale structural variations (SVs), such as deletions, insertions, translocations, inversions, duplications and extrachromosomal DNA (ecDNA), all of which have been implicated in cancer initiation and progression^24^. First reported in 1965 as double minutes (DM), ecDNA has recently been shown to be prevalent in human cancers and nearly always harbors oncogenes^25,26^. Unlike the kilobase-sized circular DNA (eccDNA) found in healthy somatic tissues^27^, ecDNA varies in size from dozens of kilobases to megabases, making it 100 to 1,000 times larger^28^. The ecDNA is characterized by high amplification and a circular structure^29^. Bulk Hi-C can detect SVs and ecDNAs in tumor tissues^30,31^, but it struggles to resolve tumor heterogeneity and evolution during therapy. Moreover, bulk DNA sequencing cannot distinguish ecDNAs from homogeneously staining regions (HSRs).

Here we introduce a highly scalable, generally accessible, droplet-based single-cell Hi-C method, Droplet Hi-C, which combines an in situ chromosomal conformation capture (3C) assay with commercially available droplet microfluidics, to simultaneously capture the three-dimensional genome structure from tens of thousands of individual cells in a single experiment. We showed the utility of Droplet Hi-C data in resolving cell-type-specific chromatin architecture in complex tissues such as the mouse brain. We further showed that Droplet Hi-C could be used to identify aberrant chromatin structure in cancer cells. We used Droplet Hi-C to identify ecDNA and mapped their chromatin interactome in tumor cells at single-cell resolution. Finally, we extended this method to enable the simultaneous capture of transcriptome and chromatin architecture in single cells.

## Results

### Development of Droplet Hi-C

**Fig. 1:**
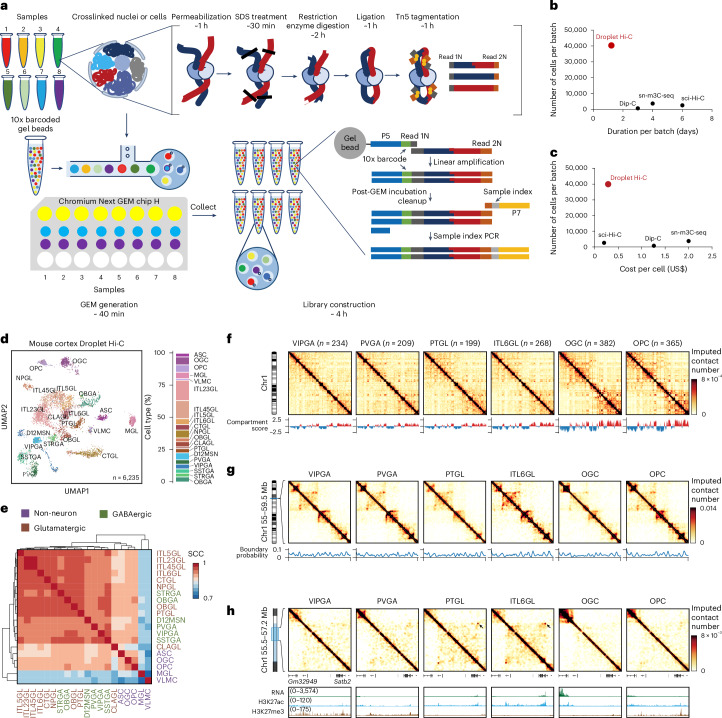
Overview and performance of Droplet Hi-C. **a**, Schematic of the Droplet Hi-C workflow. **b**,**c**, Comparison of throughput, sample preparation time (**b**) and cost (**c**) among different single-cell Hi-C methods. **d**, UMAP visualization of Droplet Hi-C data from the adult mouse cortex. ASC, astrocyte; MGL, microglia; VLMC, vascular and leptomeningeal cell; ITL45GL, intratelencephalic projecting neuron in cortical layer 4/5; CTGL, corticothalamic projection glutamatergic neuron in cortex; NPGL, near-projecting glutamatergic neuron in cortex; OBGL, glutamatergic neuron in anterior olfactory nucleus; CLAGL, glutamatergic neuron in claustrum; D12MSN, D1/D2-type medium spiny neuron; SSTGA, *Sst*+ GABAergic neuron; STRGA, GABAergic neuron in striatum; OBGA, GABAergic neuron in olfactory bulb. **e**, Genome-wide SCCs among compartment scores from different cell types. **f**, Cell-type-specific Droplet Hi-C contact maps from chr1 and compartment score at 100 kb resolution. **g**, Cell-type-specific Droplet Hi-C contact maps and boundary probabilities of the example region (chr1: 55–59.5 Mb) at 25 kb resolution. **h**, Cell-type-specific Droplet Hi-C contact maps surrounding gene *Satb2* (chr1: 55.5–57.2 Mb) at 10 kb resolution, along with a genome browser view showing transcriptome and histone modification profiles in the same cell types from the public datasets. Source data

Droplet Hi-C is based upon an in situ Hi-C procedure^32^. It captures spatial proximity genome-wide between chromatin fibers in formaldehyde-cross-linked cells or nuclei through restriction digestion and ligation in situ (Fig. 1a). After SDS treatment to remove histone proteins, DNA fragmentation and capture is then carried out in a commercially available microfluidic platform (that is, the 10x Genomics single cell assay for transposase-accessible chromatin (ATAC) kit), in which cell-specific DNA barcodes are added to the DNA fragments. This is followed by sequencing library construction and next-generation sequencing (Fig. 1a). The whole procedure lasts about 10 h from fixed cells or nuclei to final sequencing libraries, and 8 samples can be processed in parallel, enabling the profiling of 40,000 or more cells simultaneously (Fig. 1a–c). The throughput of Droplet Hi-C surpasses plate-based single-cell Hi-C methods by an order of magnitude, offering the shortest experimental duration and hands-on time to date (Fig. 1b). Given the widespread use of commercial microfluidic systems, ease of the experimental procedure and relatively low cost (Fig. 1c), Droplet Hi-C has the potential to be rapidly adopted by the research community.

**Extended Data Fig. 1:**
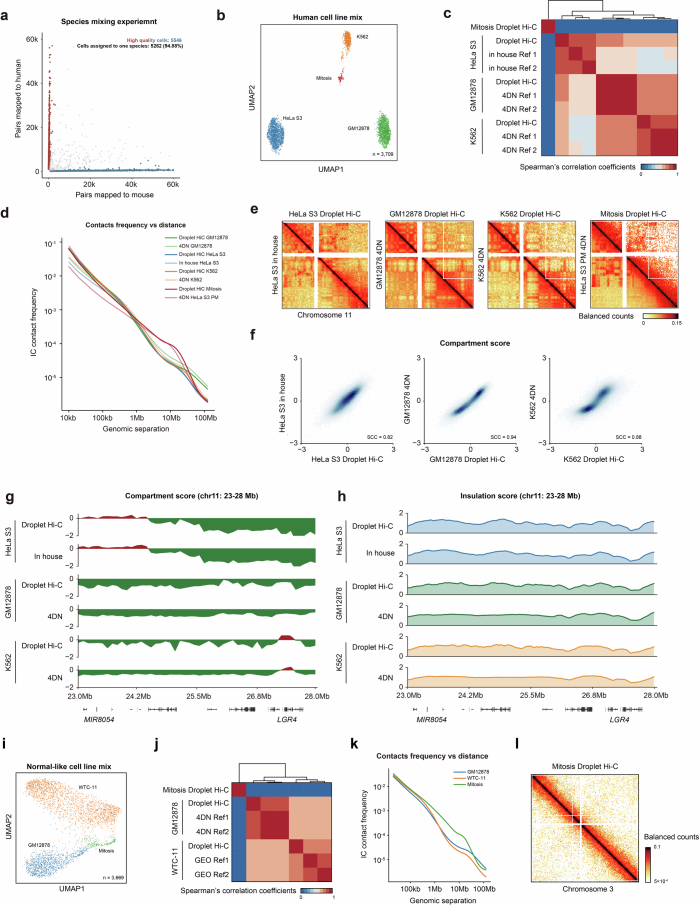
Comparison between Droplet Hi-C and *in situ* Hi-C on cultured cells. **a**, Scatter plot showing proportion of human and mouse DNA reads in each cell in the Droplet Hi-C species mixing experiment. **b**, UMAP visualization of Droplet Hi-C data on three human cell line (HeLa S3, GM12878 and K562) mixing sample. **c**, Genome-wide Spearman’s correlation coefficients of compartment score between pseudo-bulk Droplet Hi-C data and bulk *in-situ* Hi-C datasets. **d**, Comparison of contact frequency by distance between Droplet Hi-C and *in situ* Hi-C datasets of HeLa S3, GM12878, K562 and mitotic cells. **e**, Comparison of contact maps between Droplet Hi-C and bulk *in situ* Hi-C for HeLa S3, GM12878, K562 and mitotic cells on chromosome 11 at 100-kb resolution. **f**, Scatter plot showing compartment scores between Droplet Hi-C and *in situ* Hi-C for HeLa S3, GM12878 and K562 cells. Spearman’s correlation coefficients (SSC) are also shown. **g-h**, Comparison of compartment score **(g)** and insulation score **(h)** profiles on the example region (chr11: 23-28 Mb) from Droplet Hi-C and *in situ* Hi-C datasets for HeLa S3, GM12878 and K562 cells. **i**, UMAP visualization of Droplet Hi-C data from the cell line mixing experiment with two normal-like human cell lines (GM12878 and WTC-11). **j**, Genome-wide Spearman’s correlation coefficients of compartment score between Droplet Hi-C data with reference bulk Hi-C data. **k**, Contact frequency by distance from pseudo-bulk Droplet Hi-C profiles in three identified clusters. **l**, Droplet Hi-C pseudo-bulk contact map on chromosome 3 for mitosis population. Source data

To test the performance of Droplet Hi-C, we first applied it to a mixture of the human HeLa S3 cell line and the mouse embryonic stem cell (mESC) line. Equal numbers of cells from the two cell lines were combined after cross-linking and subjected to Droplet Hi-C. The results showed that 1,773 human and 3,489 mouse high-quality cells (with a total of >1,000 read pairs) were recovered after shallow sequencing, along with 284 potential doublets (Extended Data Fig. 1a). We further carried out Droplet Hi-C with a mixture of three human cell lines (K562, GM12878 and HeLa S3). After quality control and clustering using Higashi^33^, we obtained 3,709 high-quality cells with a median of 108,439 unique read pairs in each cell (duplication rate, 23.9%), including 1,604 HeLa S3 cells (median, 101,460 read pairs), 1,359 GM12878 cells (median, 109,518 read pairs), 606 K562 cells (median, 111,255 read pairs) and 140 cells with chromatin interaction patterns reminiscent of mitotic chromosomes (median, 119,573 read pairs; Extended Data Fig. 1b and Supplementary Table 1). By performing genome-wide correlation analysis with bulk in situ Hi-C references, each distinct population can be confidently assigned to one cell type (Extended Data Fig. 1c–f). The mitotic population is determined based on the similar contact frequency decay by distance pattern compared with HeLa S3 cells arrested at prometaphase^34^, and the signature contact maps enriched with frequent interactions along the diagonal (Extended Data Fig. 1d,e). On the chromosome level, cell-type-specific chromatin structures can be observed, such as the distinct Hi-C pattern of chromosomal rearrangements on chromosome (chr) 11 in HeLa S3 cells (Extended Data Fig. 1e). When focusing on a region encompassing the K562-specific upregulated gene *LGR4* (ref. ^35^), we observed an active A compartment at the *LGR4* genomic locus that is specific to the K562 cells (Extended Data Fig. 1g). The differences in compartment scores as well as insulation scores among different cell lines can also be faithfully reproduced as in the published datasets (Extended Data Fig. 1g,h). We also performed Droplet Hi-C on a mixture of diploid cells (GM12878 and WTC-11) to show that single-cell analysis of chromatin conformation enables separating different cells with normal karyotypes. Indeed, clustering of the 3,669 cells passing quality control threshold uncovered three populations (Extended Data Fig. 1i). Two of them correspond to the GM12878 cells (*n* = 2,236) and the WTC-11 cells (*n* = 1,202), based on their high correlation with bulk in situ Hi-C data (Extended Data Fig. 1j). The third population, consisting of 231 cells, appear to be cells in mitosis based on the contact maps and enrichment of long-range contacts characteristic of mitotic chromosomes^34^ (Extended Data Fig. 1k,l). In summary, these results show that Droplet Hi-C can accurately distinguish cell-type-specific chromatin organization.

### Droplet Hi-C reveals chromatin structure in mouse cortex

**Extended Data Fig. 2:**
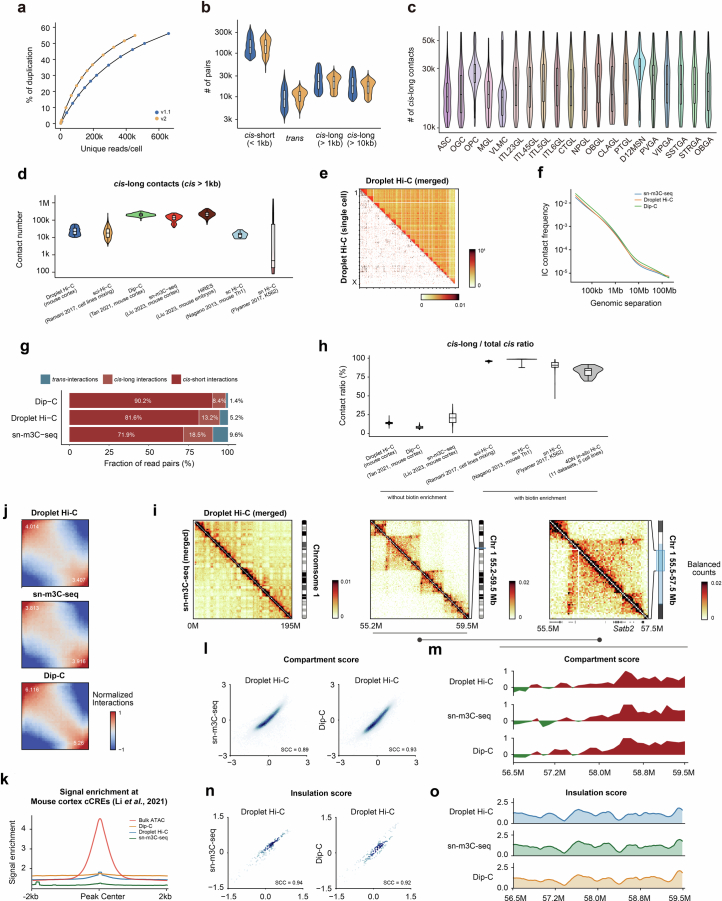
Performance metrics of Droplet Hi-C on adult mouse cortex. **a**, Comparison of library complexity by read pairs between two datasets generated with 10x Genomics Chromium Single Cell ATAC kit v1.1 and v2. n = 3,329 (v1.1), 2,906 (v2). **b**, Distribution of *cis*-short*, cis*-long or *tran* contact number per nucleus (n = 2 biologically independent experiments). **c**, Comparison of *cis*-long contact ( > 1 kb) number among different cell types (n = 2 biologically independent experiments); n = 180 (ASC), 382 (OGC), 356 (OPC), 318 (MGL), 69 (VLMC), 989 (ITL23GL), 877 (ITL45GL), 283 (ITL5GL), 268 (ITL6GL), 383 (CTGL), 183 (NPGL), 245 (OBGL), 91 (CLAGL), 199 (PTGL), 340 (D12MSN), 209 (PVGA), 234 (VIPGA), 204 (SSTGA), 184 (STRGA), 241 (OBGA). **d**, Comparison of *cis*-long contact ( > 1 kb) per cell among different single-cell Hi-C methods. n = 3,329 (Droplet Hi-C), 620 (sci-Hi-C), 250 (Dip-C), 10,000 (sn-m3C-seq), 183 (HiRes), 10 (sc Hi-C), 36 (sn Hi-C). **e**, Pseudo-bulk and representative single-cell genome-wide contact maps from mouse cortex. Color bar showed the raw contact number. **f-g**, Comparison of contact frequency by distance **(f)** and contact ratio **(g)** in mouse cortex datasets generated by Droplet Hi-C, sn-m3C-seq and Dip-C. The *cis*-long interactions in **(g)** represent contacts separated by more than 1 kb. **h**, Comparison of *cis*-long contact ratio per cell among different single-cell Hi-C methods and bulk *in situ* Hi-C. Cell number in single cell Hi-C method groups are identical as (**d**). n = 11 (4DN *in-situ* Hi-C). **i**, Comparison of multi-scale genome organization on chromosome 1 between Droplet Hi-C and sn-m3C-seq. **j**, The compartmentalization strength among Droplet Hi-C, sn-m3C-seq and Dip-C. **k**, Enrichment of sequencing reads over mouse cortex candidate *cis*-regulatory elements (cCREs) among Droplet Hi-C, sn-m3C-seq, Dip-C and bulk ATAC-seq. **l-o**, The compartment scores **(l-m)** and insulation scores **(n-o)** among Droplet Hi-C, sn-m3C-seq and Dip-C are shown. All box plot (**b, c, d, h**) hinges were drawn from the 25th to 75th percentiles, with the middle line denoting the median and whiskers denoting 2× the interquartile range. Source data

To show the feasibility of applying Droplet Hi-C to primary tissues, we used cortex tissues from 8-week-old mice and generated 6,235 high-quality single-cell chromatin profiles with a median of 175,021 unique read pairs per cell (with 22,456 *cis*-long contacts (>1 kb) and 9,954 *trans* contacts per cell, at 58% duplication rate) (Extended Data Fig. 2a–d). Despite the sparsity in contact signals, intrachromosomal interactions were enriched in the single-cell contact map (Extended Data Fig. 2e). When comparing Droplet Hi-C mouse cortex data with those of Dip-C^36^ and sn-m3C-seq^37^, decay of contact probability by distance, chromatin compartments and topologically associating domain (TAD) boundaries showed consistent patterns across methods (Extended Data Fig. 2f–o). Droplet Hi-C yields a higher *cis*-long (>1 kb) interaction ratio than Dip-C, but lower than sn-m3C-seq (Extended Data Fig. 2g,h). Although Droplet Hi-C used Tn5 transposase for chromatin fragmentation in the nucleus, it showed minimal open chromatin biases (Extended Data Fig. 2k). Overall, these results indicated that Droplet Hi-C can reliably and robustly detect chromatin structures in complex tissue.

**Extended Data Fig. 3:**
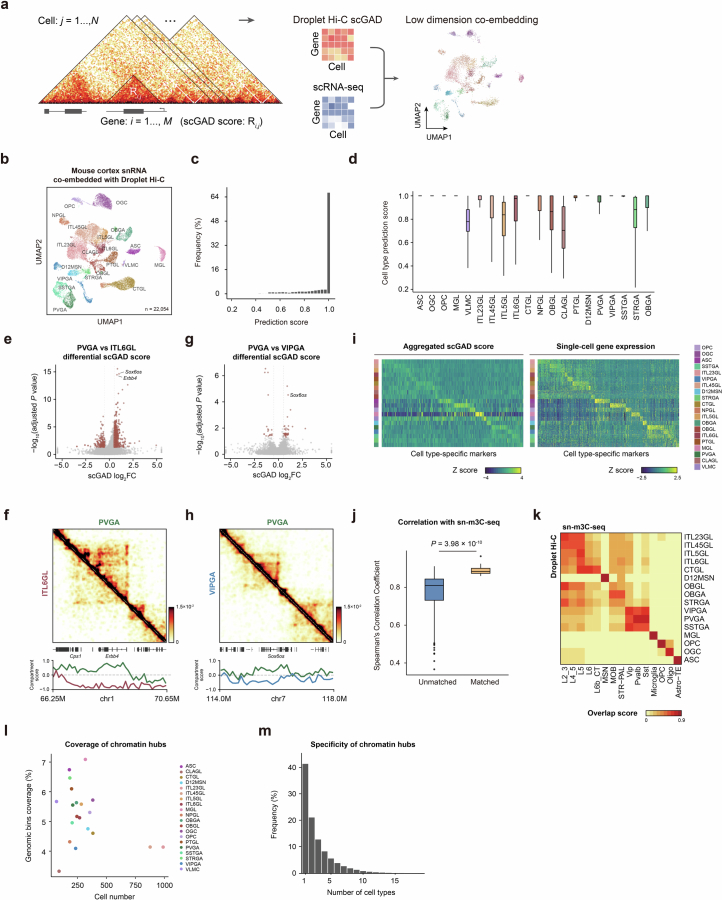
Validation of cell type identities characterized by Droplet Hi-C in mouse cortex. **a**, Schematic of the Droplet Hi-C annotation strategy using single-cell gene associating domain (scGAD) score for co-embedding. **b**, UMAP visualization of reference mouse cortex single-nucleus RNA-seq data used for co-embedding. **c**, Distribution of cell type prediction scores for Droplet Hi-C cells co-embedded with reference snRNA-seq data. **d**, Distribution of single-cell prediction scores among different annotated cell types. **e**, Volcano plot showing differential scGAD score of genes between neuronal subtypes PVGA and ITL6GL. Genes with adjusted *P* value < 0.01 and fold change > 2 are colored in red. *P* values were calculated by one-sided Wilcoxon rank-sum test. Benjamini–Hochberg FDRs were then calculated to select significant genes. **f**, Comparison of chromatin contacts between PVGA and ITL6GL at *Erbb4* (chr1: 66.25-70.65 Mb) locus. Compartment scores from the corresponding cell types are shown below the contact maps. Color bar showed the imputed contact number. **g**, Volcano plot showing the differential scGAD of genes between inhibitory neuronal subtypes PVGA and VIPGA, with significant genes colored in red. **h**, Comparison of imputed contact maps between PVGA and VIPGA around *Sox6os* gene region (chr7: 114-118 Mb) at 25-kb resolution. Compartment scores from the corresponding cell types are also shown. Color bar showed the imputed contact number. **i**, Heatmap of aggregated scGAD score at cell type-specific markers among all cell types from Droplet Hi-C data, along with single-cell gene expression heatmap of the same marker genes from snRNA-seq. **j**, Box plots showing Spearman’s correlation coefficients of compartment score between matched (n = 16) and unmatched (n = 484) cell types in Droplet Hi-C and sn-m3C-seq. *P* value was calculated by one-sided Wilcoxon rank-sum test. **k**, Heatmap showing overlap score of cell type annotations between Droplet Hi-C and sn-m3C-seq. Overlap score is calculated as the overlap between the original annotations and the joint cluster. **l**, Scatter plot showing relationship between cell number and percentage coverage of 10-kb genomic bins defined as chromatin hubs in each cell type. **m**, Specificity of identified chromatin hubs among different cell types. All box plot (**d, j**) hinges were drawn from the 25th to 75th percentiles, with the middle line denoting the median and whiskers denoting 2× the interquartile range. Source data

Chromatin interactions within the gene body have been shown to be positively associated with gene expression levels^38^, which can be used to cluster and annotate single-cell Hi-C data. We calculated the chromatin contacts on the gene body region of each gene as gene associating domain (GAD) scores^39^, and generated a cell-by-gene GAD score feature matrix. We then integrated the resulting matrix with the previous single-nuclei RNA-seq data of the mouse cortex^40^ to perform co-embedding and predict cell identity for each Droplet Hi-C cell (Extended Data Fig. 3a and Supplementary Table 2). With this strategy, we successfully resolved 20 cell groups (5 non-neuronal, 9 glutamatergic and 6 GABAergic) (Fig. 1d,e and Extended Data Fig. 3b–d). GAD score variations across neuron types aligned with the expression levels of known cell type markers (Extended Data Fig. 3e–i). As validation, we integrated Droplet Hi-C data with sn-m3C-seq data and yielded comparable cell-type annotations (Extended Data Fig. 3j,k). Chromatin organization features, such as compartments, domains and loops, were distinctly visible in the aggregated single-cell profiles (Fig. 1f–h). For example, the glutamatergic neuron marker gene *Satb2* showed specific long-range interactions with the region nearby *Gm32949* gene and had elevated expression as well as an H3K27ac mark in ITL6GL (intratelencephalic projecting neuron, cortical layer 6) and PTGL (excitatory neuron, cortex PT) glutamatergic neurons (Fig. 1h). By contrast, in other cell types, this gene remained silenced as evidenced by the presence of the repressive H3K27me3 mark. To ensure accurate chromatin organization feature analysis, we quantified the minimum number of cells required. Down-sampling mouse cortex ITL23GL (intratelencephalic projecting neuron in cortical layer 2/3) neurons showed a notable drop in compartment score and insulation score correlations below 400 cells or fewer than 10 million long-range interactions (Supplementary Fig. 1a–c), which aligns with previous studies on large chromatin features such as compartments^41^. More reads are probably needed to confidently call and analyze chromatin loops.

**Fig. 2:**
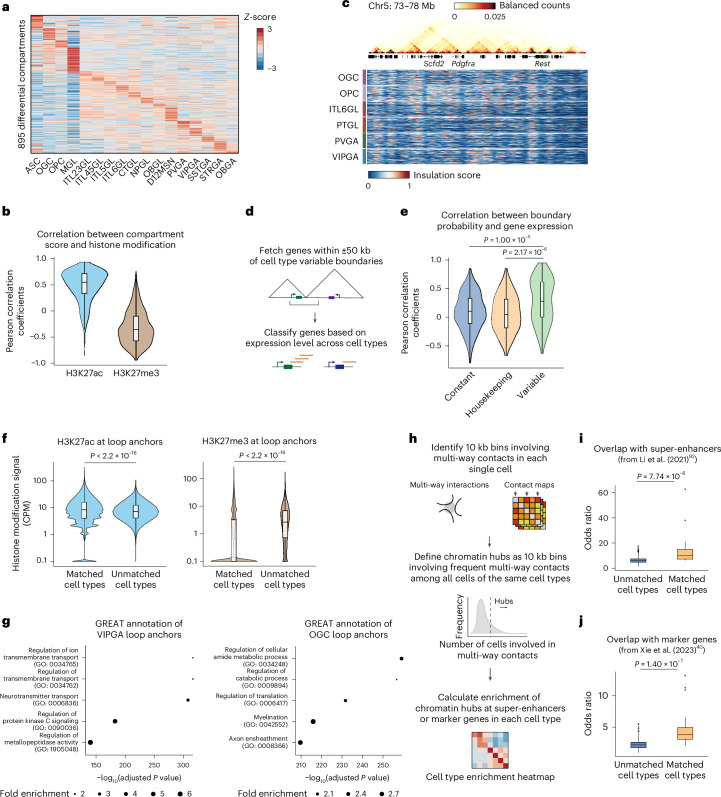
Comparative analysis of the chromatin compartments, TADs, loops and chromatin hubs across different cell types in the adult mouse cortex. **a**, Heatmap showing compartment scores of differential compartments among all cell types. **b**, Distribution of Pearson correlation coefficients between compartment scores and histone modification signals at each 100 kb bin among differential compartments; *n* = 895 (H3K27ac and H3K27me3). **c**, Comparison of single-cell insulation scores from example cell types surrounding gene *Pdgfra*. A bulk contact map at 10 kb resolution is shown above. **d**, Schematic for correlation analysis between boundary probability and nearby gene expression level. **e**, Violin plots showing Pearson correlation coefficients between gene expression level and boundary probability at variable domain boundaries among different cell types in the mouse cortex (*n* = 2 biologically independent experiments), with genes selected for clustering being excluded for analysis. Genes are classified as constant (*n* = 353), housekeeping (*n* = 479) or variable (*n* = 103) based on snRNA-seq reference and literature. *P* values were calculated by one-sided Wilcoxon rank-sum test. **f**, Comparison of histone modification signal enrichment at loop anchors among different cell types; *n* = 265,277 (all groups); *P* values were calculated by two-sided Wilcoxon signed-rank test. **g**, Top enriched GO terms for genes at loop anchors in selected cell types, VIPGA and OGC. *P* values and fold enrichment were calculated by binomial test. Benjamini–Hochberg FDRs were then calculated to select overrepresented GO terms. **h**, Diagram of the method for identifying chromatin hubs involving multi-way interactions from Droplet Hi-C data. **i**, Box plots showing the overlap between chromatin hubs and super-enhancers in matched (*n* = 16) and unmatched (*n* = 240) mouse cortical cell types. The odds ratio was calculated by Fisher exact test. *P* values were calculated by one-sided Wilcoxon signed-rank test. Data from ref. ^71^. **j**, Box plots showing the overlap between chromatin hubs and cell-type-specific marker genes in matched (*n* = 18) and unmatched (*n* = 306) cortical cell types, similar to **i** (*n* = 2 biologically independent experiments). Data from ref. ^40^. All box plot (**b**,**e**,**f**,**i**,**j**) hinges were drawn from the 25th to 75th percentiles, with the middle line denoting the median and the whiskers denoting 2 × the interquartile range. Source data

To systematically probe the differences in chromatin structure across different cell types and elucidate their relationships with chromatin state and gene expression, we first examined differences in A and B compartments at 100 kb resolution. Compartment scores correlated with transcriptional activity, with 895 genomic regions showing variable compartment scores linked to chromatin states (Fig. 2a,b). We also identified cell-type-specific TAD boundaries. For example, *Pdgfra*, which is essential for oligodendrocyte differentiation^42^, resides in an oligodendrocyte precursor cell (OPC)-specific chromatin domain boundary (Fig. 2c). We also found that genes with higher expression variation across cell types are more likely to be associated with variable TAD boundaries^33^ (Fig. 2d,e).

Next, we examined chromatin loops at 10 kb resolution. We observed that active chromatin mark H3K27ac was enriched at loop anchors in each cell type, while repressive chromatin mark H3K27me3 was relatively depleted from the loop anchors (Fig. 2f). GREAT analysis^43^ of VIPGA (CGE-derived neurogliaform cell *Vip*+)- and OGC (oligodendrocyte)-specific loop anchors showed enrichment for genes involved in neurotransmitters and myelination, respectively, corresponding to cell-type-specific functions (Fig. 2g).

Lastly, we developed an approach to identify multi-way (≥3) chromatin interactions (chromatin hubs)^44–46^ in mouse cortical cells at single-cell resolution. To do this, we first calculated all genomic loci (10 kb bins) involved in multi-way interactions in each cell as well as their frequencies among all the cells in each cell type. Next, we defined chromatin hubs as the 10 kb bins with higher-than-expected contact probability in each cell type (Fig. 2h and Methods). Around 5% of the genomic bins were identified as chromatin hubs, with most being cell type specific (Extended Data Fig. 3l,m) and enriched at super-enhancers and marker genes from the corresponding cell type (Fig. 2i,j). These findings show the ability of Droplet Hi-C to robustly analyze chromatin architecture and cell-type-specific gene regulation in complex tissues.

### Droplet Hi-C detects chromosomal aberrations in cancer cells

**Fig. 3:**
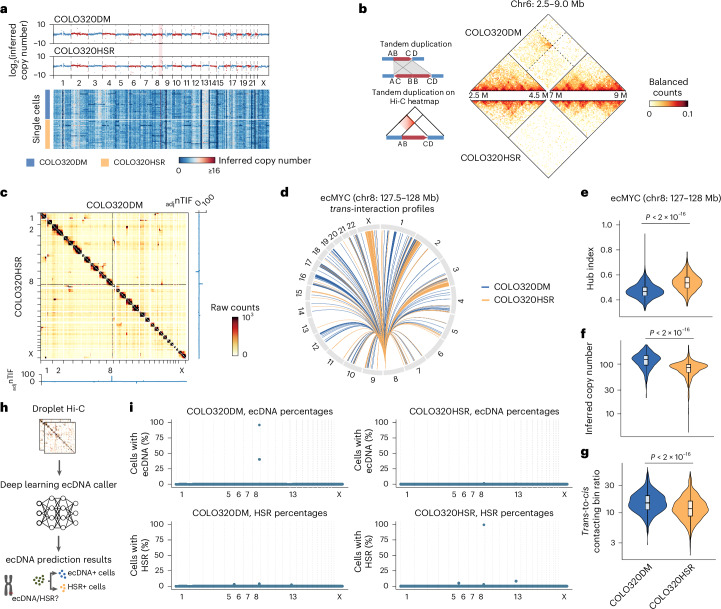
Droplet Hi-C detects SVs and ecDNAs in cancer cell lines. **a**, DNA copy numbers inferred from pseudo-bulk Droplet Hi-C profiles in COLO320DM and COLO320HSR are plotted along the genome (upper panel). The heatmap (lower panel) shows representative single-cell CNVs in each sample. The ecMYC bins in COLO320DM are highlighted in pink. **b**, Example of a sample-specific SV on chr6, predicted with EagleC. An illustration explaining the rearranged contact pattern is shown on the left. **c**, Comparison of genome-wide contact maps and _adj_nTIF between COLO320DM and COLO320HSR. **d**, A circos plot showing *trans*-interaction profiles of the genomic bin containing *MYC* in COLO320DM and COLO320HSR. **e**–**g**, Distribution of single-cell hub indexes (**e**), inferred copy numbers (**f**) and *trans*-to-*cis* contacting bin ratios (**g**) of ecMYC in COLO320DM and COLO320HSR. *n* = 1,352 (hub index, COLO320DM), 1,366 (hub index, COLO320HSR), 1,426 (inferred copy number and *trans*-to-*cis* contacting bin ratio, COLO320DM) and 1,535 (inferred copy number and *trans*-to-*cis* contacting bin ratio, COLO320HSR); *P* values are from a one-sided Wilcoxon signed-rank test. **h**, Schematic of a deep-learning-based ecDNA caller. **i**, Dot plots showing genome-wide ecDNA and HSR prediction results from the deep-learning-based ecDNA caller for COLO320DM and COLO320HSR cells. All box plot (**e**–**g**) hinges were drawn from the 25th to 75th percentiles, with the middle line denoting the median and the whiskers denoting 2 × the interquartile range. Source data

We hypothesized that single-cell Hi-C analysis could detect genomic variations in individual cancer cells and better resolve chromatin structure aberrations and ecDNA heterogeneity in them. As a proof of concept, we applied Droplet Hi-C on two colorectal cancer cell lines, COLO320DM and COLO320HSR, both carrying similar copies of the *MYC* amplicon. In COLO320DM, the *MYC* gene resides on ecDNA, whereas in COLO320HSR, *MYC* is part of HSRs. We inferred the copy number of each 1 Mb bin in individual cells and pseudo-bulk profiles, finding elevated copies of the *MYC* locus in both lines, as expected (Fig. 3a). Variations in genome-wide copy numbers were also observed (Fig. 3a). By using the inferred DNA copy number to correct bias in the contact matrices, we identified distinct SVs between these cell lines, including a duplication on chr6 (Fig. 3b).

It is challenging to differentiate *MYC* on ecDNA from HSR using whole-genome DNA sequencing alone. Tools such as AmpliconArchitect^47,48^ sometimes misclassified the HSR as a circular amplicon. Although genome-wide interaction patterns have been proposed as ecDNA indicators^49^, aggregate Hi-C profiles showed similar interchromosomal interaction patterns for the *MYC* locus in both COLO320DM and COLO320HSR (Fig. 3c). Adjusted normalized interchromosomal interaction frequency (_adj_nTIF), a method for identifying ecDNA^49^, also failed to distinguish the two forms of *MYC* amplicons (Fig. 3c).

**Extended Data Fig. 4:**
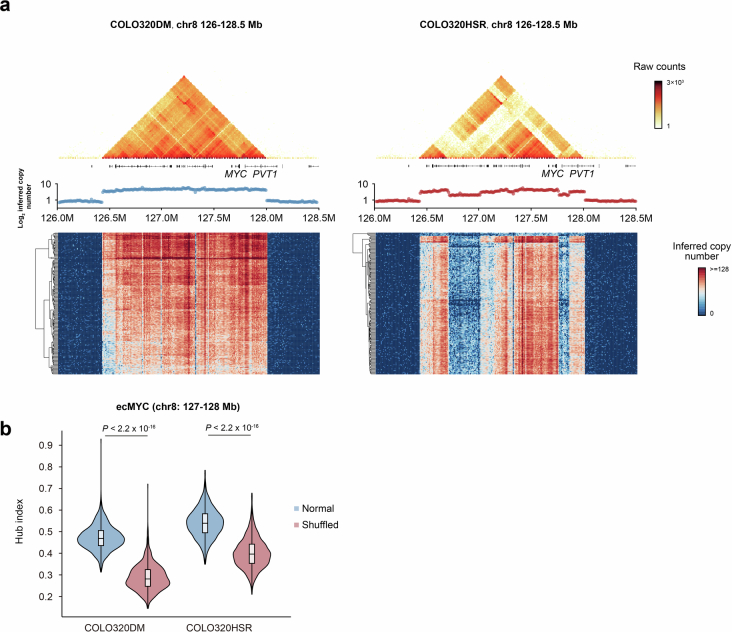
Droplet Hi-C illuminates ecDNA and HSR differences in cancer cells. **a**, Contact maps of the region containing ecMYC in COLO320DM and COLO320HSR. Inferred copy number from pseudo-bulk profiles as well as representative single cells are shown below. **b**, Single-cell hub index calculated with normal and shuffle contact profiles in COLO320DM and COLO320HSR. N = 1,352 (COLO320DM, normal), 1,359 (COLO320DM, shuffled), 1,366 (COLO320HSR, normal), 1,374 (COLO320HSR, shuffled). *P* values are calculated from one-sided Wilcoxon signed-rank test. Box plot (**b**) hinges were drawn from the 25th to 75th percentiles, with the middle line denoting the median and whiskers denoting 2× the interquartile range. Source data

We hypothesized that ecDNAs and HSRs show distinct three-dimensional genome architecture, which can be captured by single-cell Hi-C. In COLO320DM, we observed uniform interactions within ecDNA (Extended Data Fig. 4a), while COLO320HSR showed two deletions uniformly present in all cells (Extended Data Fig. 4a). Interaction hotspots for ecDNA and HSR across the genome (Methods) revealed that *MYC* ecDNA interacted more evenly with other chromosomes, while *MYC* HSR favored certain chromosomes (Fig. 3d). To quantify interchromosomal contact uniformity, we devised the hub index, which is the Gini coefficient of interchromosomal contacts for the 1 Mb *MYC* bin in each single cell. *MYC* ecDNA showed a significantly lower hub index than *MYC* HSR, indicating that ecDNAs are more evenly distributed (Fig. 3e). We also inferred MYC copy numbers (Fig. 3f) and measured the *trans*-to-*cis* contacting ratios (Fig. 3g), both showing significant differences between COLO320DM and COLO320HSR (Fig. 3f,g). COLO320DM had approximately 47% more *MYC* copies, consistent with a previous report^50^ (Fig. 3f), and a higher *trans*-to-*cis* contacting ratio than COLO320HSR (Fig. 3g). These findings suggest that ecDNA and HSR show distinct chromosomal interaction patterns in individual cells and across cell populations, further supporting that single-cell Hi-C data could be used to distinguish ecDNAs from HSRs.

To investigate whether ecDNAs cluster into ‘hubs’, we shuffled the interchromosomal contacts to generate a uniform background distribution and found that COLO320HSR had a significantly higher hub index, consistent with the tandem repeat aggregation of HSR (Extended Data Fig. 4b). Although COLO320DM had a lower hub index, it was higher than the random background, suggesting that ecDNA tends to aggregate into hubs as well^44^ (Extended Data Fig. 4b).

**Extended Data Fig. 5:**
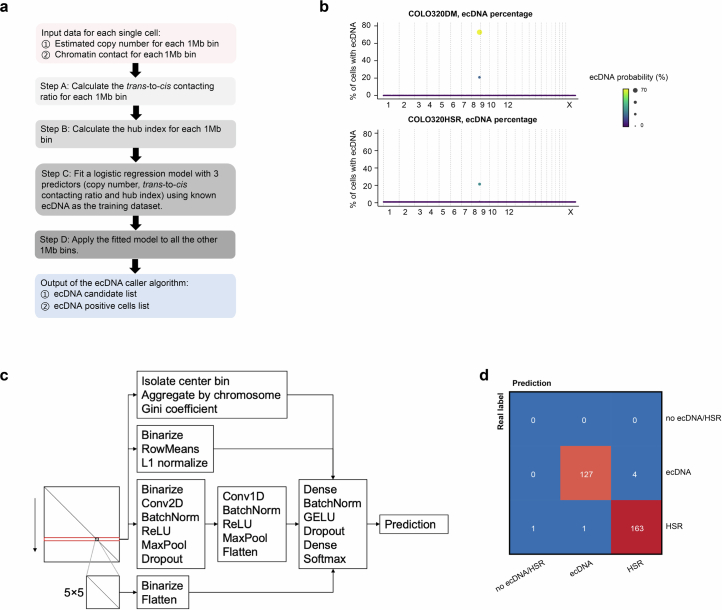
Flowchart of ecDNA caller algorithms. **a**, Workflow of multivariate logistic regression model-based ecDNA caller used to predict ecDNA identity for each genomic region and cells containing the candidate ecDNA. **b**, ecDNA prediction results from the logistic regression model-based ecDNA caller in COLO320DM and COLO320HSR. **c**, Schematic of deep learning-based ecDNA caller used to predict HSR/ecDNA identity of genomic regions, and cells containing the candidate HSR/ecDNA. **d**, Confusion matrix of prediction results versus original labels from deep learning-based ecDNA caller on COLO320DM and COLO320HSR validation datasets. Source data

These differences in chromosomal interaction patterns provide a way to distinguish ecDNA from HSR using single-cell Hi-C data. To this end, we developed a multivariate logistic regression model to predict ecDNA based on the hub index, estimated copy number and *trans*-to-*cis* contact ratios (Extended Data Fig. 5a and Methods). We trained the model to classify *MYC* (chr8: 127–128 Mb) as ecDNA in COLO320DM and HSR in COLO320HSR cells. The model achieved 0.99 specificity, 0.97 precision, 0.89 accuracy and 0.71 sensitivity in *MYC* ecDNA prediction. Applying the model genome-wide, we identified two consecutive bins (chr8: 126–128 Mb) as ecDNA in COLO320DM, with 72.7% of cells predicted to be ecDNA positive, compared with only 20.5% in COLO320HSR cells (Extended Data Fig. 5b). In a control mouse cortex dataset, no ecDNA was detected. To enhance the accuracy of ecDNA identification in single-cell Hi-C data, we developed a deep learning model using convolutional neural networks (Fig. 3h, Extended Data Fig. 5c and Methods). This model achieved 0.80 sensitivity, 0.99 specificity, 0.93 accuracy and 0.99 precision in *MYC* ecDNA prediction (Extended Data Fig. 5d). Applying this deep learning model, we identified *MYC* ecDNA in 96% of COLO320DM cells and HSR in 99.54% of COLO320HSR cells (Fig. 3i and Supplementary Table 3). Given its superior performance, we used the deep-learning-based ecDNA caller model for all our subsequent analyses.

### Droplet Hi-C uncovers ecDNA heterogeneity and evolution

**Fig. 4:**
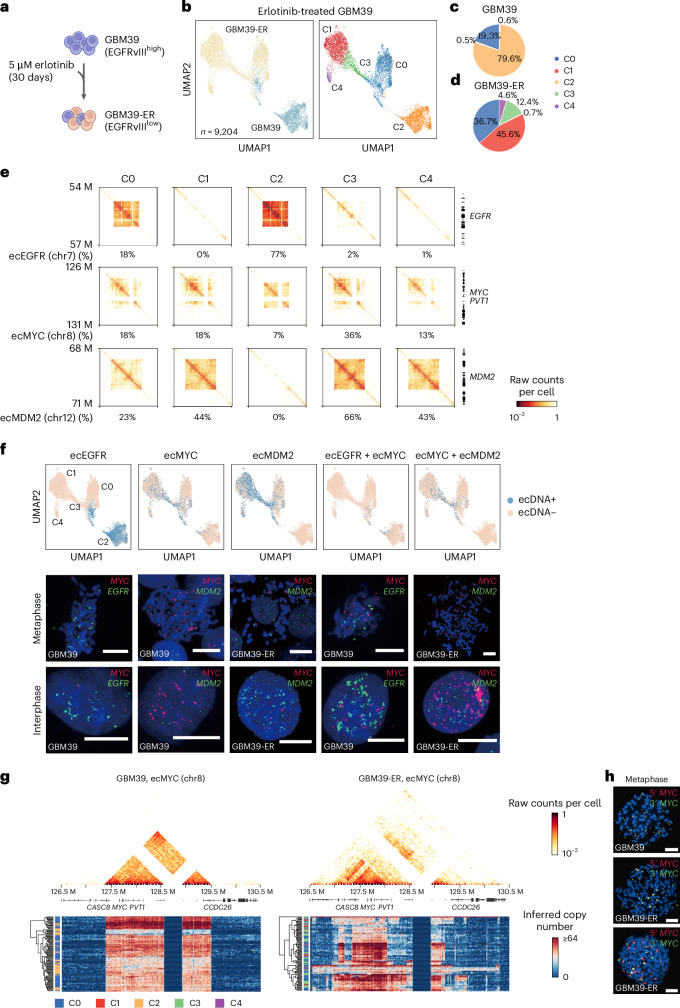
Droplet Hi-C reveals heterogeneity and evolution of ecDNAs in the GBM cell line before and after drug treatment. **a**, Illustration of the erlotinib treatment procedure for GBM39 cells. **b**, UMAP embedding visualization and clustering analysis of Droplet Hi-C data from GBM39 before and after erlotinib treatment. **c**,**d**, Pie charts showing the cell proportion of each cluster in GBM39 (**c**) and GBM39-ER (**d**). **e**, Comparison of contact maps and percentages of ecDNA-positive cells among all clusters for ecEGFR, ecMYC and ecMDM2. Genomic coordinates for key oncogenes are shown on the right. **f**, UMAP embedding visualization of ecDNA-positive and ecDNA-negative cells in GBM39 and GBM39-ER. DNA FISH of the indicated ecDNA combinations in metaphase and interphase cells is shown as validation (*n* = 2 biologically independent experiments). The ecDNA species are specified on top of the UMAP plots. Scale bars, 10 μm. **g**, Pseudo-bulk contact maps at 10 kb resolution showing the ecMYC local structure in both GBM39 and GBM39-ER samples. Heatmaps representing representative single-cell inferred CNVs in the same genomic range are shown below. **h**, DNA FISH with probes targeting in 5′ or 3′ regions of the *MYC* gene in GBM39 and GBM39-ER. Scale bars, 10 μm. Source data

**Extended Data Fig. 6:**
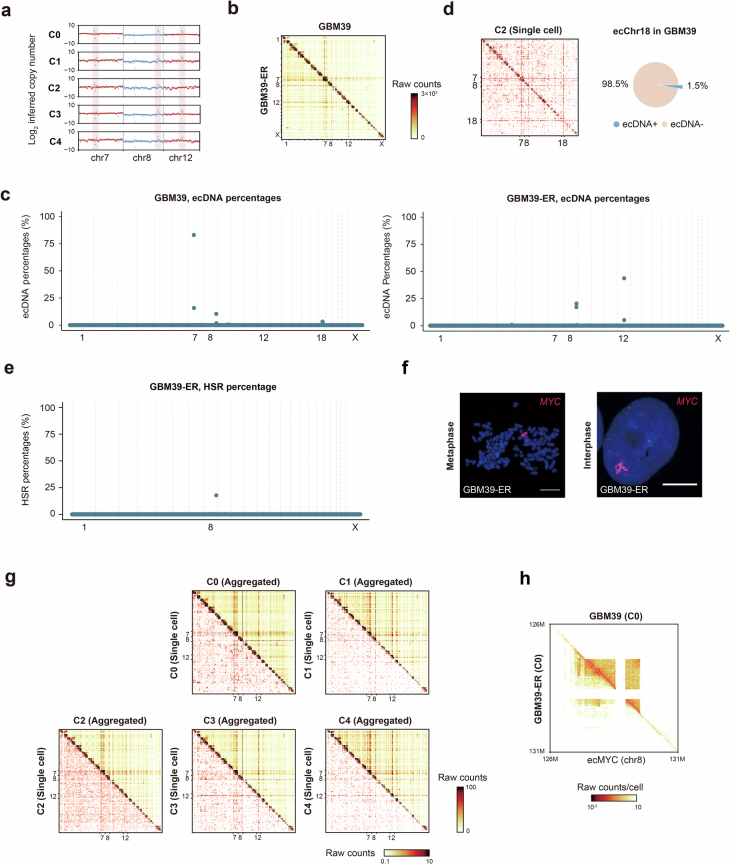
Dynamics of ecDNA in GBM39 under erlotinib treatment. **a**, Scatter plots showing inferred copy number on chromosomes containing ecDNA candidates (chromosome 7, 8, 12) among all the clusters in GBM39 and GBM39-ER. EcDNA candidate regions are highlighted in pink. **b**, Genome-wide contact maps for GBM39 and GBM39-ER cells. **c**, Scatter plots showing genome-wide ecDNA prediction results from deep learning-based ecDNA caller for GBM39 and GBM39-ER. **d**, Representative single-cell contact map and percentage of cells containing a rare ecDNA (ecChr18) found by deep learning-based ecDNA caller. **e**, Scatter plot showing HSR prediction results in GBM39-ER with the deep learning-based ecDNA caller. The 1-Mb genomic bin overlap with *MYC* gene on chr8 is predicted to be HSR. **f**, *MYC* HSR is validated by DNA FISH in GBM39-ER metaphase and interphase cells (n = 2 biologically independent experiments). Scale bar: 10 μm. **g**, Genome-wide contact maps at cluster and representative single-cell levels. **h**, Comparison of ecMYC interaction profiles for ecDNA positive cells within C0 from GBM39 and GBM39-ER. Source data

Previous studies showed that glioblastoma (GBM) cells carrying the extrachromosomal oncogenic variant of the epidermal growth factor receptor (EGFRvIII) can develop drug resistance after treatment with tyrosine kinase inhibitors such as erlotinib (ER), which coincides with the loss of *EGFR* ecDNA^51^. To further characterize the dynamics of *EGFR* ecDNA abundance and heterogeneity during the acquisition of drug resistance, we used Droplet Hi-C to profile both untreated GBM39 cells and GBM39 cells under more than 30 days of erlotinib treatment (GBM39-ER) (Fig. 4a). With 9,204 cells profiled, we resolved five different clusters of cells based on the chromatin architecture (Fig. 4b and Extended Data Fig. 6a). After erlotinib treatment, a notable evolutionary shift was observed in the GBM39 cells. Subpopulation cluster 2 (C2) nearly vanished, subpopulation cluster 0 (C0) expanded and several new subpopulations including clusters 1, 3 and 4 (C1, C3 and C4) emerged (Fig. 4b–d).

At the sample level, elevated interchromosomal contacts were observed at the *EGFR* locus and, surprisingly, the *MYC* locus before treatment. After erlotinib treatment, interchromosomal contacts at the *EGFR* locus greatly diminished, but interchromosomal contacts at the *MYC* locus increased, along with the emergence of interchromosomal contacts at a new locus on chr12 encompassing *MDM2* (Extended Data Fig. 6b). We applied the ecDNA caller to the Droplet Hi-C data to identify candidate ecDNAs in both GBM39 and GBM39-ER cells (Supplementary Table 3). In GBM39, we detected both *EGFR* ecDNA (ecEGFR) and *MYC* ecDNA (ecMYC) (Extended Data Fig. 6c, left). We also detected a low-frequency ecDNA on chr18 (1.5%, ecChr18) (Extended Data Fig. 6d). In GBM39-ER cells, we discovered ecDNA harboring the *MDM2* locus (ecMDM2) and also ecMYC (Extended Data Fig. 6c, right). In addition, we identified *MYC* HSR in GBM39-ER cells by ecDNA caller (Extended Data Fig. 6e) and validated it using fluorescence in situ hybridization (FISH) (Extended Data Fig. 6f).

At the cluster level, we calculated the percentage of cells containing each ecDNA in every cluster, revealing the dynamics of ecDNA-harboring cancer cell populations during development of drug resistance (Fig. 4e). Specifically, ecEGFR disappeared nearly completely after erlotinib treatment (C1, C3, C4) as previously reported^51^, whereas ecMDM2 appeared only in erlotinib-resistant cells and was dominant in GBM39-ER subgroup C3. In addition, ecMYC showed low frequency in GBM39 subgroup C2 and increased in transition state clusters C0 and C3. We also plotted the contact maps at 25 kb resolution for these three ecDNAs in every cluster. We observed that the structure and size of the ecMYC amplicon were altered, unlike ecEGFR and ecMDM2, suggesting a more rapid transformation at the ecMYC during drug treatment (Fig. 4e).

We also found coexistence of distinct ecDNA species in some cancer cells (Fig. 4f, top, and Extended Data Fig. 6g). In C0, a small population of cells (4.7%) harbored both ecMYC and ecEGFR, which can be validated by FISH imaging (Fig. 4f, bottom). These cells may potentially become drug resistant during erlotinib treatment, given that the gain of ecMYC may increase cellular fitness. To explore this hypothesis, we focused on the ecMYC and characterized its internal structure in GBM39 cells before and after erlotinib treatment by plotting the inferred copy number in each single cell, as the simultaneous increase in copy number can indicate that two adjacent genomic regions are co-present in the same ecDNA. Although the copy number varied among different cells, the boundaries of ecMYC in GBM39 were almost identical (Fig. 4g, left). After treatment, the ecMYC boundaries as well as the internal structure showed huge variability at single-cell resolution (Fig. 4g, right). When performing DNA FISH with a dual-color *MYC* break-apart probe set in GBM39-ER cells, while some cells showed co-amplification of both 5′ *MYC* and 3′ *MYC*, many cells showed only 5′ *MYC* amplification (Fig. 4h), which also indicated that ecMYC boundaries showed intricate transformations in GBM39-ER cells. Boundary variations in ecMYC did not show specificity to any cluster (Fig. 4g). We next selected the same number of cells from GBM39 and GBM39-ER within the transition cluster (C0) and compared the aggregated contact maps at ecMYC regions to find an intermediate ecMYC structure. Both contact maps retained their resemblance to the GBM39 and GBM39-ER cells rather than showing a similar intermediate structure, despite being from the same cluster (Extended Data Fig. 6h). Therefore, we reasoned that for GBM39 cells, erlotinib treatment probably induced the formation of a unique cell population containing distinct ecMYC, rather than selectively enriching a pre-existing cell population harboring ecMYC with growth advantage upon treatment.

### Droplet Hi-C detects heterogeneity in primary tumor samples

**Fig. 5:**
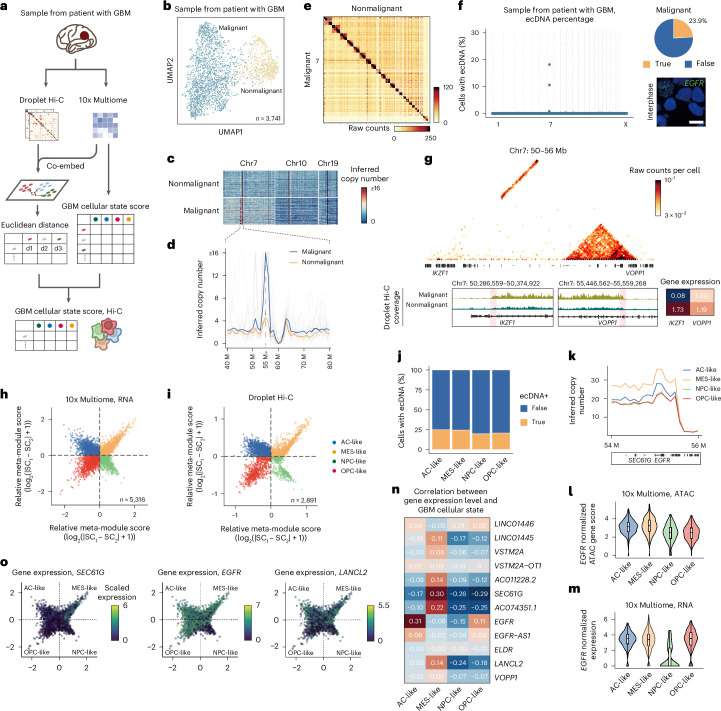
Droplet Hi-C reveals intra-tumoral heterogeneity and ecDNA in a primary glioblastoma sample. **a**, Schematic of GBM cellular state analysis for Droplet Hi-C data by co-embedding with reference 10x Genomics Multiome data. **b**, UMAP visualization of Droplet Hi-C data on the sample from the patient with GBM. **c**, Representative single-cell inferred CNVs on chr7, chr10 and chr19 in malignant and nonmalignant populations identified from Droplet Hi-C. **d**, Line plot showing copy numbers among single cells in malignant and nonmalignant populations. The colored lines highlight the median profiles. The single-cell copy number profile examples are in gray (*n* = 20). **e**, Genome-wide contact maps from malignant and nonmalignant populations. The color bars show the raw contact numbers. **f**, The ecDNA prediction results of the sample from the patient with GBM. The percentage of cells predicted to contain ecEGFR in the malignant population is shown in a pie chart. An interphase DNA FISH image of *EGFR* shows cells harboring ecEGFR in the GBM sample (*n* = 2 technically independent experiments). Scale bar, 10 μm. **g**, An example of malignant population-specific SV. A genome browser view showing the Droplet Hi-C read coverage at associated genes shown below. Predicted breakpoints of SV are highlighted in pink. The gene expression level (RPKM) of associated genes in different populations from 10x Genomics Multiome is also shown. **h**,**i**, Two-dimensional representation of cellular states based on scRNA-seq data from 10x Genomics Multiome (**h**) and Droplet Hi-C (**i**) data. Each quadrant corresponds to one cellular state. **j**, The percentages of ecEGFR-positive cells among the four different GBM cellular states. *n* = 563 (AC-like), 600 (MES-like), 247 (NPC-like) and 346 (OPC-like). **k**, Inferred copy number at 100 kb resolution of regions on ecEGFR among different GBM cellular states. **l**,**m**, Violin plots showing *EGFR* ATAC gene scores (**l**) and *EGFR* gene expression levels (**m**) among the four different GBM cellular states from the 10x Genomics Multiome dataset. Only cells with GBM cellular state scores that pass the cutoff (±0.5) are shown. *n* = 244 (AC-like), 410 (MES-like), 125 (NPC-like) and 263 (OPC-like). **n**, Heatmap showing SCCs between GBM cellular state scores and gene expression levels for all the genes located on ecEGFR. **o**, Two-dimensional representation of the GBM cellular states, colored by the expression levels of indicated genes on ecDNA. All box plot (**l**,**m**) hinges were drawn from the 25th to 75th percentiles, with the middle line denoting the median and the whiskers denoting 2 × the interquartile range. Source data

**Extended Data Fig. 7:**
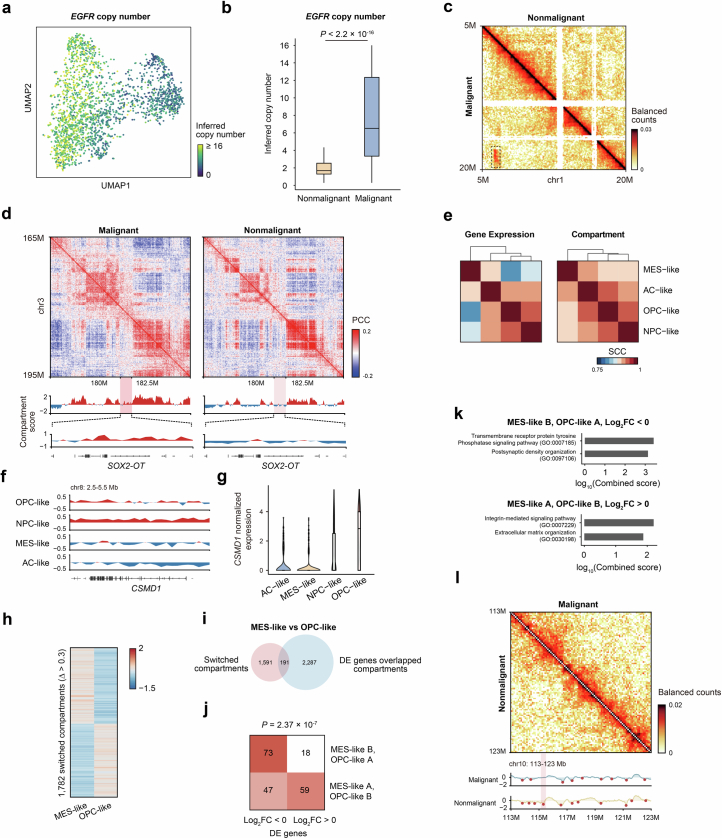
Intra-tumoral heterogeneity of chromatin architecture in a primary GBM sample. **a**, UMAP visualization of GBM Droplet-HiC data. Pseudo color of each cell indicates the inferred copy number of the 1-Mb genomic bin (chr7: 55-56 Mb) harboring *EGFR*. **b**, Box plots showing distribution of single-cell inferred copy number of 1-Mb genomic bin harboring *EGFR* in malignant or nonmalignant populations. n = 581 (Nonmalignant), 1,704 (Malignant). *P* values are calculated from one-sided Wilcoxon signed-rank test. **c**, An example of malignant population-specific SV on chromosome 1 from the GBM sample. SV is highlighted with black rectangle. **d**, Comparison of A/B compartment profiles on chromosome 3 between malignant cells and nonmalignant populations. **e**, Heatmap showing Spearman’s correlation coefficients (SCC) of 10x Genomics Multiome gene expression profiles and Droplet Hi-C compartment scores among different cellular states. **f**, Compartment profiles of *CSMD1* gene region (chr8: 2.5-5.5 Mb) in different GBM cellular states. **g**, Normalized expression of *CSMD1* in different GBM cellular states. **h**, Heatmap showing switched compartments between MES-like and OPC-like populations. **i**, Venn diagram of overlap between switched compartments and compartments with differentially expressed (DE) genes between MES-like and OPC-like populations. **j**, Enrichment of differentially expressed genes in switched compartments from (**i**). *P* value is calculated by Fisher exact test. **k**, Gene Ontology (GO) analysis of differentially expressed genes overlapped with switched compartments in MES-like and OPC-like populations using Enrichr. Combined score is determined as the product of the Fisher exact test *P* value and the Z-score of the deviation from the expected rank. **l**, Comparison of balanced contact maps and insulation profiles in an example region (chr10: 113-123 Mb) between malignant and nonmalignant populations. Example region with differential insulation score is highlighted in pink. All box plot (**b, g**) hinges were drawn from the 25th to 75th percentiles, with the middle line denoting the median and whiskers denoting 2× the interquartile range. Source data

The ecDNA is not only a biomarker for poor prognosis, but also a crucial driver in GBM^52^. Droplet Hi-C has successfully revealed ecDNA dynamics in GBM39 cells treated with drug. We further applied it to an isocitrate dehydrogenase-wildtype GBM tumor sample (Fig. 5a), clustering cells based on chromatin structure and segregating malignant from nonmalignant cells (Fig. 5b). In malignant cells, we detected expected chromosomal aberrations, such as deletions in chr10 and amplifications in chr7 (Fig. 5c), and the *EGFR* locus identified as ecDNA-like based on chromatin contact patterns and copy number variations (CNVs) (Fig. 5c–e and Extended Data Fig. 7a,b). Our ecDNA caller confirmed enrichment of ecEGFR in malignant cells (Fig. 5f and Supplementary Table 3). DNA FISH on frozen tissue sections further showed dispersed *EGFR* amplicons in the interphase nuclei (Fig. 5f, right bottom). Droplet Hi-C also identified SVs specific to the malignant population (Fig. 5g and Extended Data Fig. 7c), including translocation of the tumor suppressor gene *IKZF1* to ecEGFR without its promoter, leading to its decreased transcription (Fig. 5g). The *VOPP1* locus on ecDNA showed the strongest interaction with *IKZF1*, suggesting their fusion, but it had no discernible effect on *VOPP1* gene transcription (Fig. 5g). We observed notable changes in chromatin architecture in malignant cells at the compartment level, correlating with gene expression variations (Extended Data Fig. 7d). For example, a compartment switch from an inactive B to an active A compartment at the *SOX2-OT* locus coincided with increased transcription in the malignant cells. Droplet Hi-C effectively detected CNVs, ecDNAs, SVs and chromatin architecture specific to malignant cells in the primary GBM sample.

Previous studies using single-cell RNA-seq identified four major cellular states in malignant glioma cells: neural progenitor-like (NPC-like), OPC-like, astrocyte-like (AC-like) and mesenchymal-like (MES-like). However, the chromatin structure heterogeneity underlying these cell states remains unclear^53^. To further recapitulate different cellular states and identify intra-tumoral heterogeneity of chromatin architecture, we used k-nearest neighbors to assign malignant cells to one of the four GBM cellular states based on co-embedding with single-nucleus RNA-seq (snRNA-seq) data generated from the same sample (Fig. 5h,i). Correlation analysis using compartment scores showed a similar cell state hierarchy compared with transcriptome data and previous studies, in which the MES-like state showed higher similarity to the AC-like state, while the OPC-like and NPC-like states were more similar (Extended Data Fig. 7e)^54^.

Chromatin compartment analysis showed that state-specific gene expression aligned with chromatin architecture. For example, metastasis suppressor *CSMD1* was in an active compartment in the OPC-like and NPC-like states and in an inactive compartment in the MES-like and AC-like states, mirroring gene expression differences observed from snRNA-seq data (Extended Data Fig. 7f,g). Comparing compartment differences between MES-like and OPC-like, we identified 1,782 switched compartments (Extended Data Fig. 7h), which are significantly associated with differential gene expression (Extended Data Fig. 7i,j) and state-specific biological functions (Extended Data Fig. 7k). In addition to compartment changes, we also observed insulation score alterations and loss of domain boundaries in malignant cells (Extended Data Fig. 7l). Such disruption can rewire enhancer–promoter interactions, contributing to the GBM progression^55^.

We also characterized ecDNA heterogeneity across different GBM cellular states. While the fraction of cells containing ecEGFR was similar across states (Fig. 5j), AC- and MES-like cells had higher ecEGFR copy numbers (Fig. 5k), corresponding to increased *EGFR* gene expression and chromatin accessibility (Fig. 5l,m). Furthermore, by defining ecDNA boundary, we found that expression levels of genes on the ecDNA varied with cellular states, and that such correlation could not be fully explained by ecDNA copy number, indicating a complex regulatory mechanism governing ecDNA genes expression among cellular states (Fig. 5n). Consistent with previous reports^53^, the expression level of *EGFR* gene showed the highest correlation with AC-like state scores (Fig. 5n). Other genes on the ecEGFR, such as *SEC61G* and *LANCL2*, showed preferential expression in the MES-like state, or the AC- and MES-like states, respectively (Fig. 5o).

**Extended Data Fig. 8:**
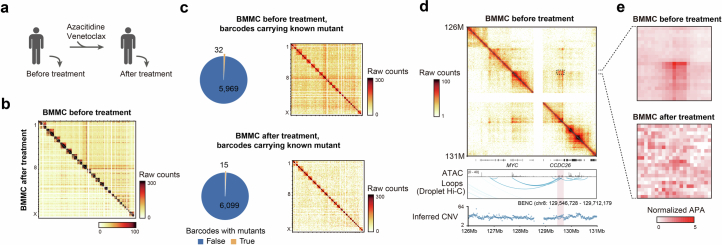
Droplet Hi-C reveals chromatin architecture changes in a BMMC patient before and after treatment. **a**, Illustration of the treatment procedure and sample collection. **b**, Pseudo-bulk genome-wide contact maps for BMMC samples from patient before and after treatment. **c**, Proportions of cells containing known tumor-associated mutations in BMMC samples before and after treatment. Aggregated genome-wide contact maps from mutant-carrying cells are also shown. **d-e**, Contact map showing a chromatin loop between *MYC* and its enhancer region (BENC) on ecMYC before treatment. Normalized aggregate peak analysis (APA) scores of before- and after-treated samples are shown aside.

To further show the utility of Droplet Hi-C in clinical tumor samples, we applied it to bone marrow mononuclear cells from a patient with myelodysplastic syndrome and secondary acute myeloid leukemia (AML) before and after treatment with azacitidine (a DNA methyltransferase inhibitor) and venetoclax (an inhibitor of the anti-apoptotic protein BCL2) (Extended Data Fig. 8a). Pretreatment bone marrow mononuclear cells harbored a large (~5 MB) ecDNA containing the *MYC* gene. After treatment, the ecMYC disappeared (Extended Data Fig. 8b,c), consistent with the patient’s remission. Besides, the proportion of malignant cells also diminished greatly after treatment (Extended Data Fig. 8c). The long-range interactions between *MYC* and blood enhancer cluster, which activate *MYC* expression in AML^56^, were detected pretreatment but disappeared after therapy, suggesting a role in tumor progression (Extended Data Fig. 8d,e). Thus, in this patient’s tumor, ecDNA appears to promote *MYC* expression not only through increased DNA copy numbers but also through cancer-specific enhancer–promoter interactions.

In summary, Droplet Hi-C enables the detection of ecDNA dynamics in tumors before and after treatment. Beyond ecDNA, it also identifies SVs and chromatin architecture, shedding light on the regulatory programs driving tumor progression and drug resistance. Droplet Hi-C offers substantial potential for understanding tumor evolution and treatment response.

### Joint profiling of chromatin architecture and transcriptome

**Fig. 6:**
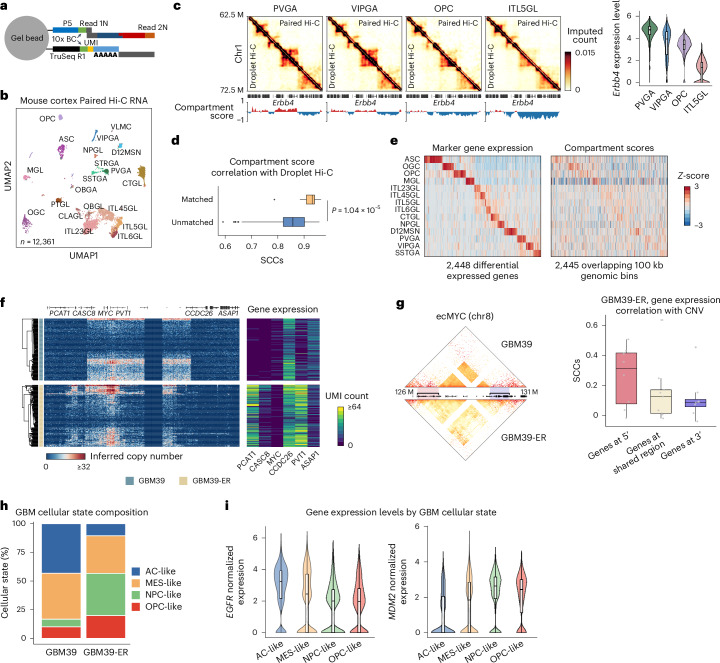
Joint profiling of chromatin architecture and transcriptome in single cells with Paired Hi-C. **a**, Schematic of the Paired Hi-C molecule barcoding step with the 10x Genomics Multiome kit. BC, cell barcode. **b**, UMAP visualization of mouse cortex Paired Hi-C RNA-seq data. **c**, Comparison of pseudo-bulk contact maps between Droplet Hi-C and Paired Hi-C at the region surrounding gene *Erbb4*, along with compartment score profiles from Paired Hi-C in representative cell types (*n* = 3 biologically independent experiments). The color bar shows the imputed contact number. Violin plots of *Erbb4* expression levels in representative cell types are also shown. **d**, Box plots showing SCCs of compartment scores between matched and unmatched cell types in Droplet Hi-C and Paired Hi-C. *n* = 15 (matched) and 225 (unmatched). *P* values were calculated by one-sided Wilcoxon signed-rank test. **e**, Heatmaps showing marker gene expression levels and compartment scores of corresponding 100 kb bins among all cell types. **f**, Single-cell inferred copy number heatmaps of the region (chr8: 126.5–130.5 Mb) harboring ecMYC in GBM39 and GBM39-ER. Single-cell UMI count heatmaps of representative genes on ecMYC are also shown. **g**, SCCs between gene expression levels and copy numbers for ecMYC genes in 5′ or 3′ variable regions or shared regions in GBM39-ER are shown. An illustration of ecMYC variable regions is shown on the left. *n* = 9 (5′ gene), 10 (shared) and 11 (3′ gene). **h**, Comparison of cell proportions of the four GBM cellular states between GBM39 and GBM39-ER samples. **i**, Comparison of expression levels of *EGFR* and *MDM2* among the four GBM cellular states. *n* = 4,307 (AC-like), 6,791 (MES-like), 4,945 (NPC-like) and 3,123 (OPC-like). All box plot (**c**,**d**,**g**,**i**) hinges were drawn from the 25th to 75th percentiles, with the middle line denoting the median and the whiskers denoting 2 × the interquartile range. Source data

Single-cell joint profiling of chromatin organization and transcriptome helps investigate the relationships between gene expression and chromatin architecture. We modified the Droplet Hi-C protocol to work with the 10x Genomics Chromium Single Cell Multiome kit, creating Paired Hi-C, which simultaneously profiles RNA and Hi-C from the same single nuclei (Fig. 6a and Supplementary Fig. 2). This protocol uses milder SDS treatment to reduce RNA degradation and a lower formaldehyde concentration for better complexity of chromatin contact profiles and revised the cDNA library preparation to recover more cDNA molecules. Paired Hi-C offers higher throughput and efficiency compared with other microwell- or combinatorial-indexing-based single-cell joint Hi-C and RNA-seq assays (Supplementary Fig. 2).

**Extended Data Fig. 9:**
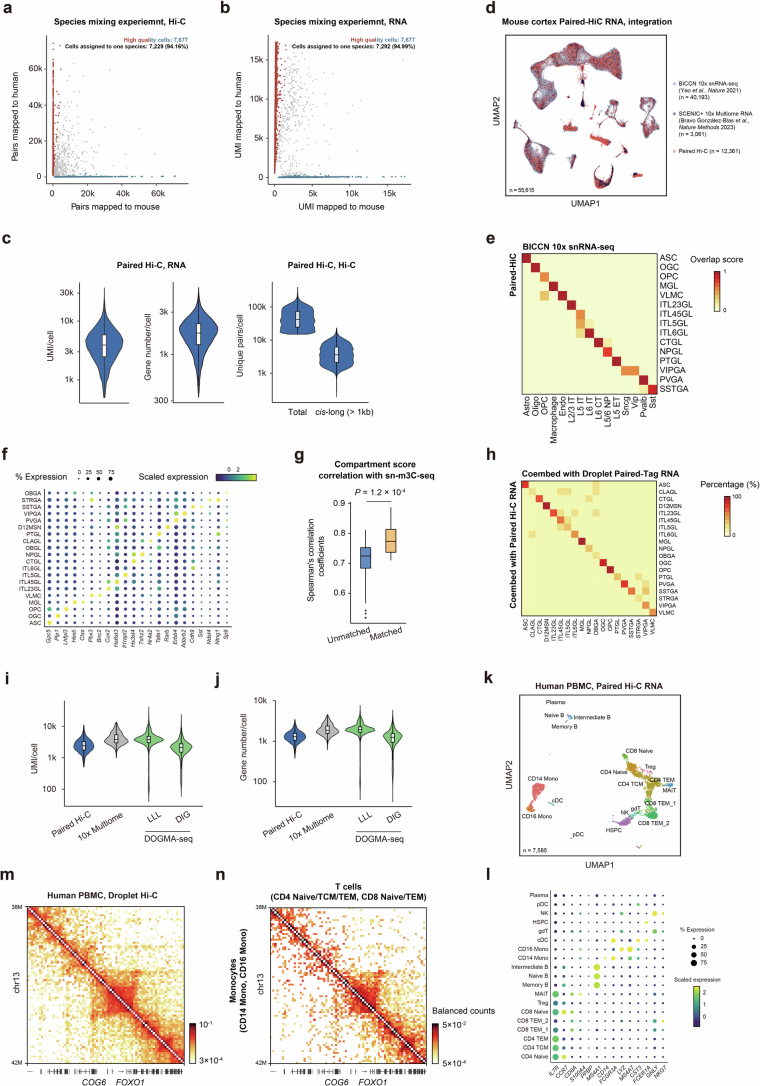
Joint analysis of chromatin architecture and transcriptome using Paired Hi-C. **a**, Scatter plot showing proportion of human and mouse Hi-C read pairs in each cell from Paired Hi-C species mixing experiment. **b**, Scatter plot showing proportion of human and mouse RNA UMI in each cell from Paired Hi-C species mixing experiment. **c**, Distribution of UMI, gene number, and contacts per nucleus in Paired Hi-C data from adult mouse cortex (n = 3 biologically independent experiments). n = 12,360. **d**, UMAP visualization of single-nucleus transcriptome profiles from Paired Hi-C experiment co-embedded with the reference BICCN and SCENIC+ datasets. **e**, The overlap scores of shared annotations between BICCN single-nucleus RNA-seq dataset and Paired Hi-C single-nucleus RNA-seq dataset. **f**, Dot plot showing expression level of marker genes in each cell type. **g**, Box plots showing Spearman’s correlation coefficients of compartment score between matched and unmatched cell types in sn-m3C-seq and Paired Hi-C. n = 241 (Unmatched), 15 (Matched). *P* value is calculated from one-sided Wilcoxon signed-rank test. **h**, Overlap of predicted annotations when using Droplet Paired-Tag snRNA-seq dataset as reference versus using Paired Hi-C snRNA-seq dataset as reference to co-embed Droplet Hi-C data. **i**, Violin plots showing UMI per cell distribution in Paired Hi-C RNA modality, compared to 10x Genomics Multiome or DOGMA-seq under different conditions. LLL (low-loss lysis) cell lysis condition; DIG (digitonin) lysis condition. n = 7,585 (Paired Hi-C), 9,480 (10x Genomics Multiome), 8,591 (LLL), 11,868 (DIG). **j**, Violin plots showing gene number per cell distribution in Paired Hi-C RNA modality versus other methods. **k**, UMAP visualization of human PBMC Paired Hi-C data clustered and annotated based on the transcriptome profiles. **l**, Dot plot showing scaled expression of marker genes across 19 predicted human PBMCs cell types in Paired Hi-C dataset. **m**, Balanced pseudo-bulk contact map of all PBMC cells on chromosome 3 at 100-kb resolution. **n**, Pseudo-bulk contact maps of aggregated T cells versus monocytes at the same region as in (**m**). Cell types used to aggregate are shown. All box plot (**c, g, i, j**) hinges were drawn from the 25th to 75th percentiles, with the middle line denoting the median and whiskers denoting 2× the interquartile range. Source data

We first validated Paired Hi-C by performing a species-mixing experiment with HeLa S3 and mESC, successfully separating human and mouse cells based on Hi-C (HeLa S3: 3,928, mESC: 3,301, total: 7,677) and RNA (HeLa S3: 3,999, mESC: 3,293, total: 7,677) data (Extended Data Fig. 9a,b). Applying Paired Hi-C to the adult mouse cortex sample, we obtained 12,361 joint single-cell profiles with 42,210 read pairs per cell for the Hi-C modality (duplication rate, 66.2%), and a median of 3,914 unique molecular identifiers (UMI) per cell and 1,746 genes per cell for the RNA modality (duplication rate, 74%; Extended Data Fig. 9c). We successfully identified 20 cell types using the Paired Hi-C RNA data, including 9 excitatory neuron types, 6 inhibitory neuron types and 5 non-neuronal types (Fig. 6b and Supplementary Table 4). Cell-type annotation was validated by integration with other snRNA-seq or single-cell RNA-seq (scRNA-seq) datasets and expression patterns of marker genes (Extended Data Fig. 9d–f).

Although single-cell three-dimensional genome features were relatively limited in Paired Hi-C, compartment scores strongly correlated with Droplet Hi-C and sn-m3C-seq profiles from the same cell types (Fig. 6c,d and Extended Data Fig. 9g). We detected concordant changes of compartment and gene expression at cell-type-specific marker genes (Fig. 6c–e). For example, *Erbb4* gene was in the A compartment in PVGA (MGE-derived neurogliaform cell *Pvalb*+) and VIPGA GABAergic neurons, but they were placed in the B compartment in OPC non-neuronal types and ITL5GL (intratelencephalic projecting neuron, cortical layer 5) glutamatergic neurons, correlating with higher *Erbb4* expression in GABAergic neurons (Fig. 6c). We also used transcriptome information from Paired Hi-C and co-embedded it with Droplet Hi-C. The results agree well with the cell annotations based on the Droplet Paired-Tag^40^ (Extended Data Fig. 9h). As such, we can augment the number of chromatin contacts for each cell type by combining Paired Hi-C and Droplet Hi-C.

We also applied Paired Hi-C to human peripheral blood mononuclear cells (PBMCs) and obtained 7,585 high-quality single-cell profiles. The transcriptome data were benchmarked against other methods such as 10x Genomics Multiome and DOGMA-seq^57^, showing comparable performance (Extended Data Fig. 9i,j). Using the 10x Genomics Multiome RNA as the reference, we performed label transfer to predict cell types in the Paired Hi-C RNA modality, and filtered out cells whose identities could not be accurately assigned (Extended Data Fig. 9k). Marker gene expression profiles confirmed the accuracy of annotation (Extended Data Fig. 9l). We performed co-embedding of Droplet Hi-C with Paired Hi-C profiles to compensate for the Hi-C sparsity in Paired Hi-C data, and analyzed the chromatin architecture differences between the T cells and monocytes. T cells showed strong chromatin loops between *COG6* and *FOXO1* gene loci, correlating with higher transcription of these genes compared with monocytes (Extended Data Fig. 9m,n).

Paired Hi-C enables joint analysis of chromatin structure and gene expression at single-cell resolution, offering insights into the regulation of gene activation or repression. We applied it to erlotinib-treated GBM39 cells to study how changes in chromatin structure, particularly ecDNA, affect gene expression. Paired Hi-C linked ecDNA structure with gene expression in single cells, showing that gene expression generally correlates with ecDNA copy number (Fig. 6f). Genes at the 5′ end of the variable ecMYC boundaries such as *CASC8* and *PCAT1* showed a strong copy number–expression correlation (Fig. 6f,g), while genes at the 3′ end, such as the *CCDC26* gene, showed high expression despite low copy numbers (Fig. 6f,g). This indicates a complex relationship between ecDNA structure and gene expression.

**Extended Data Fig. 10:**
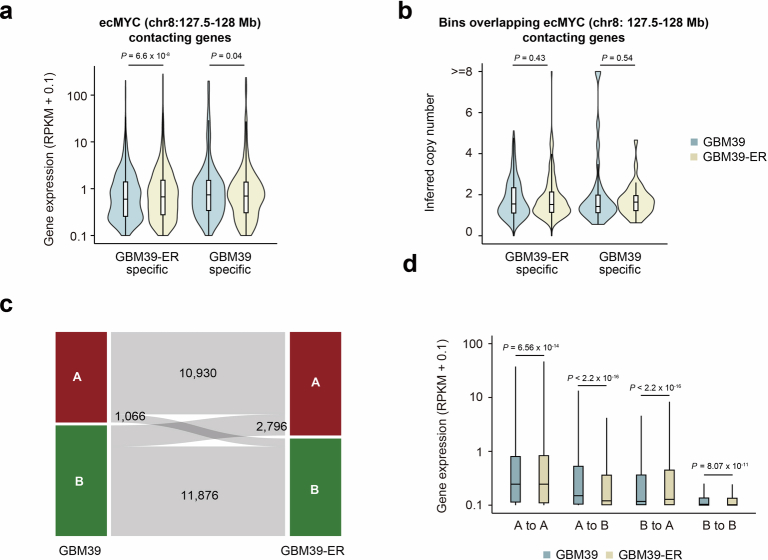
Paired Hi-C reveals alterations in gene expression associated with variations in chromatin structure. **a**, Comparison of expression levels for ecMYC *trans*-contacting genes between GBM39 and GBM39-ER cells (n = 2 biologically independent experiments). Only genes showed expression (RPKM > 0.1) in at least one groups were retained for analysis. n = 1,169 (GBM39-ER specific), 256 (GBM39 specific). *P* values are calculated from two-sided Wilcoxon signed-rank test. **b**, Violin plots showing the inferred copy number of 500-kb genomic bins at ecMYC interaction hotspots between GBM39 and GBM39-ER cells. n = 387 (GBM39-ER specific), 32 (GBM39 specific). *P* values were calculated from two-sided Wilcoxon signed-rank test. **c**, Summary of changes in A/B compartments in GBM39 and GBM39-ER sample. **d**, Expression of genes classified by the underlying A/B compartment transitions in GBM39 and GBM39-ER cells. n = 22,193 (A to A), 1,409 (A to B), 4,132 (B to A), 8,720 (B to B). *P* values were calculated from two-sided Wilcoxon signed-rank test. All box plot (**a, b, d**) hinges were drawn from the 25th to 75th percentiles, with the middle line denoting the median and whiskers denoting 2× the interquartile range. Source data

In addition, we observed different *trans*-interaction patterns of ecMYC in GBM39 and GBM39-ER cells that were associated with distinct transcriptional responses. Genes with GBM39-specific ecMYC interactions were more highly expressed in GBM39 cells, while GBM39-ER-specific ecMYC interacting genes were more highly expressed in GBM39-ER cells (Extended Data Fig. 10a). The higher expression levels were probably not caused by the elevation in the copy number of interacting regions (Extended Data Fig. 10b). This observation is consistent with a potential regulatory role of ecDNA in global transcription as suggested previously^49^.

In the sample from the patient with GBM, ecEGFR showed higher copy numbers and gene expression levels in AC-like and MES-like cells, possibly driving changes in cellular states under erlotinib treatment. We classified GBM39 and GBM39-ER cells into four GBM cellular states using Paired Hi-C RNA-seq data and found a reduction in the differentiated-like population after erlotinib treatment (Fig. 6h). As found in the GBM tumor sample, *EGFR* expression was higher in AC-like and MES-like cells (Fig. 6i). Interestingly, *MDM2* expression was enriched in the progenitor states (OPC- and NPC-like), which became more prevalent after treatment (Fig. 6i). These results highlight the unique interplay among ecDNA dynamics, cellular states and drug resistance.

Erlotinib treatment also caused dramatic shifts in chromatin architecture and gene expression changes in GBM39 cells. A total of 1,066 A compartments switched to B compartments, and 2,796 B compartments shifted to A compartments, with correlated changes in gene expression (Extended Data Fig. 10c,d). Genes in compartments that changed from A to B showed decreased expression, while those in compartments that shifted from B to A showed increased expression (Extended Data Fig. 10d).

In summary, Paired Hi-C can directly link chromatin reorganization and structural alterations, such as ecDNA, to gene expression. This tool should offer valuable insights into gene regulatory mechanisms in development and disease, particularly in understanding tumor progression and drug resistance.

## Discussion

Recent advances in single-cell Hi-C methods have greatly expanded their applications. However, existing techniques still face challenges in analyzing heterogeneous tissues and tumor biopsies. Our Droplet Hi-C method, using a commercially available microfluidic platform, provides high-throughput single-cell Hi-C assays with minimal hands-on time and lower costs. Applying Droplet Hi-C to the adult mouse cortex, we identified cell-type-specific chromatin structures and correlated them with epigenetic modifications. In addition, we detected changes in chromatin organization, CNVs, SVs and ecDNAs in cancer cells and patient tumor samples, particularly during drug treatment. We further developed Paired Hi-C, which profiles both chromatin architecture and transcriptome in single cells, enabling the study of gene expression in relation to three-dimensional genome structure. Although the Hi-C complexity in Paired Hi-C is lower than in Droplet Hi-C, combining data from both methods allowed us to resolve and annotate cell types while improving chromatin contact resolution.

Our droplet-based single-cell Hi-C methods offer advantages in scalability, speed, cost and adaptability. However, improvements are needed, particularly in the library complexity of Paired Hi-C. In addition, while Droplet Hi-C has a lower ratio of *cis*-long (>1 kb) interactions than some in situ Hi-C methods (Extended Data Fig. 2h), it performs similarly to or better than other single-cell Hi-C methods that do not enrich ligated DNA fragments (Extended Data Fig. 2h). While *cis*-short interactions (<1 kb) provide limited information on chromatin contacts, they are valuable for analyzing CNVs and SVs in cancer cells. Biotin enrichment of *cis*-long interactions could improve chromatin architecture capture efficiency and reduce sequencing costs.

The ecDNA is prevalent in cancer, often carrying oncogenes^25,26^. Current single-cell ecDNA detection methods fall into two categories: computational tools using whole-genome sequencing data from a next-generation sequencing^47^ or a third-generation sequencing platform^58,59^ and Circle-seq^60–62^, which retains circular DNA by degrading linear DNA. However, distinguishing ecDNAs from HSRs remains challenging. Here we develop an ecDNA detection algorithm that uses single-cell Hi-C data to reliably identify ecDNAs and effectively distinguish them from HSRs. Our deep-learning-based ecDNA caller can accurately detect ecDNA in single cells and calculate its proportion in the population. By analyzing intra-ecDNA contacts, we can pinpoint ecDNA boundaries. For instance, we identified ecMYC boundary variations in GBM39-ER cells.

Pan-cancer studies have revealed ecDNAs in many cancer types and their association with poor clinical outcomes^63–67^. Furthermore, tumors with ecDNA show unique therapeutic vulnerabilities^68,69^. Thus, given the independent prognostic value of ecDNA and its potential to be targeted, recent calls have been made to include ecDNA profiling in clinical specimens^70^. Our Droplet Hi-C and ecDNA caller offer a reliable toolkit detecting ecDNA in clinical samples, as shown in GBM and AML tumors. This toolkit is particularly valuable for heterogeneous samples, in which ecDNA structures and copy numbers evolve during treatment. Importantly, our ecDNA caller can distinguish between amplifications on ecDNA and HSRs, which may have important therapeutic implications. In our study, we observed ecEGFR disappearance, ecMDM2 emergence and ecMYC structural changes in GBM39 cells following treatment with erlotinib, a tyrosine kinase inhibitor commonly used in the treatment of *EGFR*-positive tumors. The poor response to erlotinib in GBM may be linked to the ecDNA evolution. Similarly, in a sample from a patient with AML, ecMYC was no longer detectable after treatment with azacitidine and venetoclax. Our methods hold promise for advancing ecDNA research in tumor diagnosis and treatment.

## Methods

### Experimental protocol for Droplet Hi-C

A brief description of the Droplet Hi-C experimental procedure is provided below.

#### Fixation

Cells were cross-linked in 1% formaldehyde, which was diluted from 37% formaldehyde with 1× PBS (pH = 7.4), and incubated at room temperature for 10 min. After cross-linking, the reactions were quenched in 200 mM glycine and incubated at room temperature for 5 min. Quenched reactions were spun down at 1,000 × *g* for 5 min at 4 °C and resuspended using 1% bovine serum albumin (BSA) in 1× PBS (pH = 7.4) to wash twice. One million cells were aliquoted into each tube. The cells were spun once again at 1,000 × *g* for 5 min, the supernatant was removed, and the pellet was flash frozen in liquid nitrogen and finally stored indefinitely at −80 °C.

#### In situ Hi-C

Cell pellets were lysed with pre-cold 300 μl lysis buffer (10 mM Tris–HCl, pH 8.0 (Thermo Fisher Scientific, 15568025), 10 mM NaCl (Sigma, S5150), 0.2% IGEPAL CA-630 (Sigma, I8896) and 1× protease inhibitor (Roche, 5056489001)) on ice for 45 min, centrifuged at 1,000 × *g* for 5 min at 4 °C to collect nuclei, and washed once with 200 μl lysis buffer. The nuclei were resuspended with 50 μl 0.5% SDS and incubated at 62 °C for 10 min on Thermomixer. Then, we added 145 μl of nuclease-free H_2_O and 25 μl 10% Triton X-100 (Sigma, 93443) to quench SDS and incubated samples at 37 °C for 15 min on Thermomixer at 300 rpm. To the samples were added 27 μl of 10× CutSmart Buffer and three restriction enzymes, including 50 U DpnII (NEB, R0543L), 62.5 U MboI (NEB, R0147M) and 7.5 U NlaIII (NEB, R0125L); then, the samples were incubated at 37 °C for 90 min on Thermomixer at 550 rpm. The enzymes were deactivated at 65 °C for 20 min and then cooled down to room temperature. The nuclei were collected by centrifugation at 1,000 × *g* for 5 min at 4 °C and washed once with 200 μl ligation buffer (100 μl 10× T4 DNA ligase buffer (NEB, B0202S), 5 μl 20 mg ml^−1^ BSA (NEB, B9000S), 865 μl H_2_O); the ligation reaction was performed with 200 μl ligation buffer and 20 μl T4 DNA ligase (NEB, M0202L) at 37 °C for 40 min on Thermomixer at 300 rpm.

#### Single-cell Hi-C sequencing library construction

The ligated nuclei pellets were resuspended in 1 ml 1% BSA in PBS (diluted from 10% BSA in PBS (Sigma, A1595) using 1× PBS (pH = 7.4)) in each tube, 1 μl 1,000× 7-AAD (Invitrogen, A1310) was added and the nuclei were sorted by fluorescence-activated nuclei sorting with an SH800 cell sorter (Sony) for the isolation of single nuclei (Supplementary Fig. 3). The nuclei were collected in collection buffer (5% BSA in PBS) at 5 °C and immediately centrifuged for 5 min at 1,000 × *g* and 4 °C to collect the nuclei. The nuclei were washed once with 1× Nuclei Buffer (diluted from 20× Nuclei Buffer (10x Genomics, PN-2000207)), resuspended in 10 μl 1× Nuclei Buffer and counted on an RWD C100-Pro fluorescence cell counter with DAPI staining. According to the desired targeted nuclei recovery number, 3,000–15,000 nuclei were aliquoted into PCR tubes, to which was added Transposition Mix following the user’s guide for the Chromium Next GEM Single Cell ATAC Reagent Kits v1.1 or the Chromium Next GEM Single Cell ATAC Reagent Kits v2 (10x Genomics). The incubation time for tagmentation is 60 min for both the v1.1 and v2 kits. We also extended the index PCR elongation time from 20 s to 1 min. The double-sided size selection was changed to 1.14× SPRIselect to remove only small fragments.

All sequencing experiments were performed with an Illumina NextSeq 2000 or NovaSeq 6000 sequencer.

### Experimental protocol for Paired Hi-C

A brief description of the Paired Hi-C experimental procedure is provided below.

#### Fixation

Cells were cross-linked in 0.6% formaldehyde, which was diluted from 37% formaldehyde with 1× PBS (pH = 7.4), and incubated at room temperature for 10 min. After cross-linking, the reactions were quenched in 200 mM glycine and incubated at room temperature for 5 min. The quenched reactions were spun down at 1,000 *g* for 5 min at 4 °C and resuspended using 1% BSA in 1× PBS (pH = 7.4) to wash twice, and then 1 million cells were aliquoted into each tube. The cells were spun once again at 1,000 × *g* for 5 min, the supernatant was removed, and the pellet was flash frozen in liquid nitrogen and finally stored indefinitely at −80 °C.

#### In situ Hi-C

Cell pellets were lysed with pre-cold 300 μl lysis buffer (10 mM Tris–HCl (pH 8.0; Thermo Fisher Scientific, 15568025), 10 mM NaCl (Sigma, S5150), 0.2% IGEPAL CA-630 (Sigma, I8896), 1× protease inhibitor (Roche, 5056489001), 1 U μl^−1^ RNaseOUT (Invitrogen, 10777-019) and 1 U μl^−1^ SUPERaseIn inhibitor (Invitrogen, AM2694)) on ice for 45 min, then centrifuged at 1,000 × g for 5 min at 4 °C to collect nuclei and washed once with 200 μl lysis buffer. The nuclei were resuspended with 50 μl 0.5% SDS, which was diluted from 10% SDS using 1× PBS (pH = 7.4) with 1 U μl^−1^ RNaseOUT and 1 U μl^−1^ SUPERaseIn inhibitor, and incubated at 37 °C for 60 min on Thermomixer at 300 rpm. Then, we added 197 μl quenching buffer (137.59 μl of nuclease-free H_2_O, 25 μl 10% Triton X-100, 27 μl 10× CutSmart, 1 U μl^−1^ RNaseOUT and 1 U μl^−1^ SUPERaseIn inhibitor) and incubated samples at 37 °C for 15 min on Thermomixer at 300 rpm. The samples were centrifuged at 1,000 × *g* for 5 min at room temperature, the supernatant was removed and the pellets were resuspended with 250 μl digestion buffer (1× CutSmart Buffer, 1 U μl^−1^ RNaseOUT, 1 U μl^−1^ SUPERaseIn inhibitor, 50 U DpnII, 62.5 U MboI (NEB, R0147M) and 5 U NlaIII), and then incubated at 37 °C for 90 min on Thermomixer at 550 rpm. The enzymes were deactivated at 65 °C for 20 min and then cooled to room temperature. The nuclei were collected by centrifugation at 1,000 × *g* for 5 min at 4 °C and washed once with 200 μl ligation buffer (827.5 μl H_2_O, 100 μl 10× T4 DNA ligase buffer, 5 μl 20 mg ml^−1^ BSA, 25 μl SUPERaseIn inhibitor and 12.5 μl RNaseOUT), and the ligation reaction was performed with 200 μl ligation buffer and 20 μl T4 DNA ligase at 37 °C for 40 min on Thermomixer at 300 rpm.

PBMCs (Allcells, LP, CR, MNC, 100 M) were cross-linked in 1% formaldehyde. Then, 1 million cell pellets were lysed with 200 μl of high-salt lysis buffer 1 (50 mM HEPES (pH 7.4), 2 mM EDTA (pH 8.0), 140 mM NaCl, 0.25% Triton X-100, 0.5% IGEPAL CA-630, 10% glycerol and 1× proteinase inhibitor cocktail) and incubated on ice for 10 min. After this, the cells were pelleted at 500 *g* for 2 min at 4 °C and then resuspended in 200 μl of high-salt lysis buffer 2 (10 mM Tris–HCl (pH 8), 3 mM EDTA, 200 mM NaCl, 1× proteinase inhibitor cocktail). The solution was incubated on ice for 10 min. Following this, the cells were then pelleted at 500 *g* for 2 min at 4 °C and then resuspended in 200 μl of 1× T4 DNA ligase buffer containing 0.2% SDS. The cells were then incubated at room temperature for 1 h. To quench the reaction, 200 μl of 1× T4 DNA ligase buffer and 10 μl 10% Triton X-100 were added to the tube. Finally, the cells were spun at 500 *g* for 4 min at 22 °C. The pellets were resuspended with 250 μl digestion buffer (1× T4 DNA ligase buffer, 1 U μl^−1^ RNaseOUT, 1 U μl^−1^ SUPERaseIn inhibitor, 80 U DpnII) and then incubated at room temperature for 2 h. The nuclei were collected by centrifugation at 500 *g* for 4 min at 4 °C and washed once with 200 μl ligation buffer (857.5 μl H_2_O, 100 μl 10× T4 DNA ligase buffer, 5 μl 20 mg ml^−1^ BSA, 25 μl SUPERaseIn inhibitor and 12.5 μl RNaseOUT), and the ligation reaction was performed with 200 μl ligation buffer and 20 μl T4 DNA ligase at room temperature for 40 min.

#### Single-cell sequencing library construction

The ligated nuclei pellets were resuspended in 1 ml 1% BSA in PBS (diluted from 10% BSA in PBS using 1× PBS (pH = 7.4)) with 1 U μl^−1^ RNaseOUT and 1 U μl^−1^ SUPERaseIn inhibitor in each tube, 1 μl 1,000× 7-AAD (Invitrogen, A1310) was added, and the nuclei were sorted by fluorescence-activated nuclei sorting with an SH800 cell sorter (Sony) for the isolation of single nuclei. The nuclei were collected in collection buffer (5% BSA in PBS with 5 U μl^−1^ Recombinant RNasin (Promega, PAN2515)) at 5 °C and immediately centrifuged for 5 min at 1,000 × *g* and 4 °C to collect the nuclei. The nuclei were washed once with 1× Nuclei Buffer (diluted from 20× Nuclei Buffer (10x Genomics, PN-2000207) with 1 mM DTT and 1 U μl^−1^ Recombinant RNasin), resuspended in 10 μl 1× Nuclei Buffer and counted on an RWD C100-Pro fluorescence cell counter with DAPI staining. According to the desired targeted nuclei recovery number, 3,000–15,000 nuclei were aliquoted into PCR tubes, to which was added Transposition Mix following the user’s guide of Chromium Next GEM Single Cell Multiome ATAC + Gene Expression (10x Genomics). The tagmented nuclei were loaded onto a Chromium Next GEM Chip J for droplet generation with a Chromium X microfluidic system (10x Genomics). The gel bead-in-emulsions (GEMs) were collected, reverse transcription and cell barcoding were performed using a thermal cycler and the reaction was quenched following the user’s guide of the Chromium Next GEM Single Cell Multiome ATAC + Gene Expression (10x Genomics). After GEM cleanup and pre-amplification, the SPRIselect products were eluted into 100 μl elution buffer (Qiagen, 19086) instead of 160 μl. For scHi-C library construction, we extended the index PCR elongation time from 20 s to 1 min, and the double-sided size selection was changed to 1.55× SPRIselect to remove only small fragments. For scRNA-seq library construction, we used 0.8× SPRIselect to purify cDNA twice instead of 0.6× SPRIselect to keep short cDNA fragments.

All sequencing experiments were performed with an Illumina NextSeq 2000 or NovaSeq 6000 sequencer.

### Processing of Droplet Hi-C data

Custom scripts for demultiplexing, mapping and extracting single-cell contacts from Droplet Hi-C data were publicly available at https://github.com/Xieeeee/Droplet-Hi-C. First, depending on the indexing primers used, Droplet Hi-C fastq files were demultiplexed using Illumina bcl2fastq (v2.19.0.316) or 10x Genomics cellranger-atac mkfastq (v2.0.0). After demultiplexing, a custom script was used to extract the cellular barcode sequence from Read2, and barcodes were aligned to the white list provided by 10x Genomics using Bowtie (v1.3.0)^72^. Aligned barcode sequences were appended to the beginning of the read name to record the cellular identity of each read. Next, sequencing adapters were detected and trimmed with Trim-Garole (v0.6.10)^73^. Cleaned reads were then mapped to the human (hg38) or mouse (mm10) reference genome using BWA-MEM (v0.7.17)^74^, with arguments ‘-SP5M’ specified. Finally, after mapping, valid contacts were parsed, sorted and deduplicated by Pairtools (v0.3.0)^75^, with barcode information stored in a separated column in the pairs file. Contacts were balanced and stored in cool format using Cooler (v0.8.10) for visualization and downstream analysis^76^. High-quality cells were selected based on the number of total unique contacts per cell in each library.

### Analysis of Droplet Hi-C data

Here we describe the general analysis strategy and workflows for our Droplet Hi-C data. Dataset-specific manipulations in the paper, if any, will be indicated.

#### Visualization of Hi-C contact matrices

Bulk, pseudo-bulk or single-cell contact matrices in cool format were visualized and plotted using Cooler along with Matplotlib (v3.5.1)^77^.

#### Signal enrichment calculation

Density plots and signal enrichment over Center for Epigenomics of the Mouse Brain Atlas candidate *cis*-regulatory elements (cCREs) were generated using bamCoverage from deepTools (v3.5.3). Peaks that overlapped with ENCODE blacklist (v2) regions were removed during the calculation of enrichment^78^.

#### Embedding and clustering of single-cell Droplet Hi-C dataset on cultured cells

We used Higashi to infer low-dimensional cell embeddings for all our Droplet Hi-C datasets without imputations, including a human cell line mix (HeLa–GM12878–K562; Extended Data Fig. 1b), GM12878–WTC-11 (Extended Data Fig. 1i), GBM39–GBM39-ER (Fig. 4b) and the sample from the patient with GBM (Fig. 5b). For visualization, the L2 norm of cell embeddings was projected to a two-dimensional space with uniform manifold approximation and projection (UMAP). To identify cells with similar identity, we performed Leiden clustering using igraph (v0.9.9) and leidenalg (v0.8.8)^79,80^.

#### Contact frequency by distance analysis

We used the ‘expected_cis’ module from cooltools (v0.5.1) to calculate the average contact frequency as a function of genomic separation^81^. To plot the curve, we performed smoothing and aggregation among chromosome regions.

#### Imputation of single-cell chromatin contacts on the adult mouse cortex Droplet Hi-C dataset

For the adult mouse cortex Droplet Hi-C data, we used the scHiCluster (v1.3.4) to perform contact matrix imputation for individual cells at three different resolutions: 100 kb (for cell embedding and visualization), 25 kb (for domain boundary calling) and 10 kb (for cell embedding and loop calling)^82^. In brief, contacts from individual cells were first masked for ENCODE blacklist regions^78^. scHiCluster then performed linear convolution and random walk with restart to impute the sparse single-cell contact matrices for each chromosome. Considering storage efficiency, we output the imputed results for the whole chromosome at 100 kb, for the 10.05 Mb window at 25 kb and for the 5.05 Mb window at 10 kb resolution for each cell. For cancer cells or tumor samples, we did not perform imputation owing to CNVs and SVs.

#### Embedding and annotation of single-cell Droplet Hi-C dataset on the adult mouse cortex

For the imputed adult mouse cortex datasets and PBMC datasets, we adopted a previously described method to co-embed Hi-C data with gene expression data (snRNA-seq) generated from the same tissue and to assign cell-type identities^39^. Briefly, single-cell GAD (scGAD) score *R*_*ij*_ is calculated as the raw number of interactions at the gene body region for gene *i* in cell *j* with the 10 kb imputed matrix. This method has been implemented in scHiCluster as the ‘gene_score’ module. By this, the single-cell chromatin interaction profiles can then be represented as a cell-by-gene scGAD score matrix, which serves as the input for integration with snRNA-seq data.

To perform integration, we began by normalizing and selecting the top 2,000 variable genes in reference snRNA-seq data. These genes were subjected to dimension reduction by principal component analysis (PCA) using Scanpy (v1.7.2), retaining 30 principal components^83^. The derived PCA model was then applied to transform the scGAD score matrix. Subsequent integration was conducted with a tailored version of Seurat integration using canonical correlation analysis^84^. For the visualization of co-embedded data, we calculated the nearest neighbor graph (*k* = 25) and UMAP embedding with Scanpy.

To annotate cell identities within the Droplet Hi-C dataset, we identified the 15 nearest snRNA-seq neighbors for each cell via PyNNDescent (v0.5.6) using Euclidean distances^85^. These distances were scaled and converted into standardized scores so that the total score is added up to 1, and the neighbor with the minimal distance would have the highest score. The identity of each Droplet Hi-C cell was inferred by nominating the cell type label that garnered the highest standardized score among its top nearest neighbors.

To validate the cell type annotations, co-embedding of Droplet Hi-C and sn-m3C-seq datasets was performed using scHiCluster, which facilitates the low-dimensional representation of chromatin contact information. For each cell, imputed matrices at 100 kb resolution were binarized and flattened. The binarized data from individual cells in both datasets were then concatenated into a larger matrix. PCA was applied to this matrix on a per-chromosome basis. The PCA results for each chromosome were concatenated for a second round of PCA to generate the final cell embeddings. Batch effects were corrected using harmonypy (v0.0.9)^86^. After batch correction, Leiden clustering was used to identify co-embedding clusters. The validity of the original annotation was verified by calculating the overlap coefficients (*O*_*i*,*j*_) between the clusters and the original annotations from Droplet Hi-C (*A*) and sn-m3C-seq dataset (*B*): ${O}_{i,j}=\min ([\frac{{A}_{i}\cap {C}_{k}}{{A}_{i}}],\,\max [\frac{{B}_{j}\cap {C}_{k}}{{B}_{j}}])$, where *i* indicates the query cell type from Droplet Hi-C, *j* indicates the reference cell type from sn-m3C-seq and *k* indicates the co-embedding cluster.

#### Analysis of A and B compartments

On sample levels, we used the ‘eigs_cis’ module from cooltools to perform eigen decomposition on balanced contact matrices and calculate the compartment score at 100 kb resolution^81^. The orientation of the resulting eigenvectors was adjusted to correlate positively with the CpG density of the corresponding 100 kb genomic bins, thereby determining the sign of the compartments (A and B). Similarity between samples is calculated as the Spearman’s correlation coefficient (SCC) of compartment scores among all autosomes (Extended Data Fig. 1c,j). To illustrate chromatin interactions within and between compartments, we used the ‘saddle’ module from cooltools to calculate the average observed versus expected contact frequency, categorized by compartment scores.

To generate pseudo-bulk profiles for distinct cell types or clusters within a sample, we adopted a uniform approach outlined before to minimize bias and facilitate direct comparisons. Initially, the raw contact matrices at the sample level were normalized for distance effects. Next, Pearson correlation coefficients were computed on the distance-normalized matrices, and PCA was then performed on the correlation matrix. The model fitted at the sample level was used to transform the raw contact matrices of various cell types or clusters. To compare the similarity between cell types or clusters, we calculated the SCC of the compartment score using the compartment that intersected with variable gene regions (Fig. 1e and Extended Data Fig. 7e). To identify differential compartments, we used dchic (v2.1)^87^, selecting only those with an adjusted *P* value < 0.01 and a Manhattan distance greater than the 2.5th percentile of the standard normal distribution for further downstream analysis.

For joint analysis of compartment and cell-type-specific histone modifications, we used the recently published adult mouse cortex Droplet Paired-Tag dataset^40^. We retained only cell types that contain >100 cells and were shared between datasets to ensure comparability among the analyses. For all differential compartments, we calculated the Pearson correlation coefficients between the compartment score and the signal of H3K27ac or H3K27me3 (counts per million mapped reads (CPM)) within the same 100 kb genomic bins among various cell types.

#### Analysis of chromatin domains

On sample levels, we used the ‘insulation’ module from cooltools to compute the insulation score for raw contact matrices at 25 kb resolution. To delineate cell-type-specific variable domain boundaries in mouse cortex data, we used TopDom (v0.0.2) on 25 kb resolution imputed contact matrices for each individual cell^88^. We defined the ‘boundary probability’ for a given bin as the proportion of cells within a particular cell type that designate the bin as a domain boundary. The presence or absence of a domain boundary is summarized by *n* cell types into an *n* × 2 contingency tables. We then computed the chi-square statistics and *P* value for each bin, and the domain boundaries were classified as variable if they showed a false discovery rate (FDR) <0.001 and a boundary probability difference >0.05.

For correlation analysis of gene expression surrounding variable domain boundaries, we first identified genes within a 100 kb window centered on the variable domain boundaries. We then calculated Pearson correlation coefficients to quantify the relationship between boundary probabilities and expression levels (reads per kilobase per million mapped reads (RPKM)) of these genes from Droplet Paired-Tag data among shared cell types. The genes were further categorized into ‘housekeeping’, ‘constant’ and ‘variable’. In brief, the housekeeping gene list was taken from a previous report^89^. The top 2,500 variable genes from Droplet Paired-Tag RNA modality were selected as the variable group. Genes with a similar expression level as the top 2,500 variable genes but were not within the ‘housekeeping’ or ‘variable’ group were treated as ‘constant’.

#### Analysis of chromatin loops

For loops calling on sample levels, we adopted the ‘dots’ module in cooltools, which implements the principle of the commonly used HiCCUPS loop calling strategy, to call loops on the 10 kb raw contact matrices^90^.

A modified version of the SnapHiC workflow implemented in scHiCluster is used to perform loops calling on the 10 kb imputed contact matrices for mouse cortex cell types^91^. To compare histone modification enrichment at loop anchors, we calculate H3K27ac or H3K27me3 CPM among all cell types at all loop anchors identified. When histone modification profiles and loop anchors are from the same cell types, the enrichment is classified as ‘matched’; otherwise, it is classified as ‘unmatched’.

Gene Ontology (GO) annotation of loop anchors was performed using rGREAT (v1.26.0) in R^92^. The GO biological process was selected for annotations. The result is used to generate plots in Fig. 2g.

#### Multi-way chromatin interaction analysis

Multi-way interactions are extracted with ‘pairtools parse2’ function in Pairtools, which is designed to rescue complex chromatin ligation events. After deduplication, only *trans* interactions and *cis* interactions with genomic distance >10 kb were retained. Read pairs overlapping with the ENCODE mm10 blacklist regions were removed. For each pair-end read on autosomes supporting multi-way contacts, we collected the 10 kb genomic bins containing their anchors. Finally, we defined one pair-end read to support multi-way contacts, if it contacts ≥3 unique 10 kb bins (can be both intrachromosomal and interchromosomal).

To define contact hubs for multi-way interactions in each mouse brain cell type, we followed the below strategy: first, the reference genome (mm10 for mouse cortex) was segmented into consecutive 10 kb bins. Next, a binary indicator was assigned to each 10 kb bin in each single cell, depending on whether it is involved in a multi-way contact. Then, for each 10 kb bin, we calculated the frequency of single cells involved in multi-way contacts among all cells belonging to the same cell type. We excluded the top 1% of bins with the highest frequency or bins with sequence mappability scores <0.8 when calculating the mean and standard deviation for frequency distribution, then converted the frequency distribution into a *Z*-score. Finally, a 10 kb bin is defined as in multi-way contact hub if its *Z*-score is >1.96. Custom scripts to analyze the multi-way interaction hub are available at https://github.com/HuMingLab/Multiway.hub.

#### Enrichment of chromatin features at multi-way chromatin hubs

After identifying the multi-way chromatin hubs in each mouse cortex cell type, we performed enrichment analysis against cCREs, super-enhancers and cell-type specifically expressed genes. Specifically, cCREs are calculated and converted into 10 kb genomic bins from single-nucleus ATAC using sequencing (snATAC-seq) data^71^. Super-enhancers are called using HOMER ‘findPeaks -style super’ on the identified cCREs within each cell type with default parameters^93^, and converted into 10 kb genomic bins. For each cell type *A* involving a multi-way contact hub, we calculated its overlap with cCREs or super-enhancers from another cell type (*B*) and created a 2 × 2 contingency table. We then performed Fisher’s exact test to evaluate the significance of enrichment and reported the log_2_ odds ratio as the enrichment score. We enumerated all pairs of cell types and compared the log_2_ odds ratio in matched (*AA*) versus unmatched (*AB*, where *B* ≠ *A*) cell types. We repeated the same enrichment analysis for cell-type-specific marker genes from published datasets^40^, except that we used the 10 kb bin overlap with the marker gene transcription start site for enrichment analysis.

#### Infer CNV with Droplet Hi-C data

Copy number is inferred from Hi-C data using the ‘calculate-cnv’ module in NeoLoopFinder (v0.4.3)^94^. For each single cell, the output residuals from the generalized additive model in ‘calculate-cnv’ were directly used to estimate the copy number ratio. On bulk or pseudo-bulk level, an additional hidden Markov model-based segmentation is performed using the ‘segment-cnv’ module on the copy number ratio to determine the boundaries of copy number ratio segments. We assume all samples used in this paper for CNV calculation are diploid; therefore, the inferred copy number is equal to 2 × copy number ratio. A genome-wide CNV heatmap is plotted at 10 Mb resolution, where chromosome level CNV is plotted at 1 Mb resolution, and the regional CNV profile is plotted at 10 kb resolution in R using pheatmap (v1.0.12). The inferred copy number is further used to assist in identifying ecDNA candidates and the associated significant *trans-*interactions, and to correct the bias in contact matrices for SV identification.

#### Identify SV from Droplet Hi-C data

SV was identified using the ‘predictSV’ module in EagleC (v0.1.9)^95^. In short, CNV effects on contact matrices at 5 kb, 10 kb and 50 kb resolutions were removed using the ‘correct-cnv’ module in NeoLoopFinder. ‘predictSV’ then uses a deep learning model to predict SV at each resolution on the corrected matrices and combines all results to obtain a uniform, high-resolution SV list at 5 kb resolution. The ‘annotate-gene-fusion’ module is applied on the final SV list to annotate gene fusion events.

#### Metrics to define chromatin interactions

We compiled a suite of metrics to assess the *cis-* and *trans-*interaction patterns at specific genomic loci. These metrics include the following: (1) contact evenness or ‘hub index’, quantified as the Gini coefficient for *trans-*interactions among all chromosomes excluding chromosome Y given a 1 Mb genomic bin, and was derived using ineq (v0.2-13) in R; (2) *trans*-to-*cis* contacting bin ratio (*R*_*i*_), which compares the quantity of interacting bins within the same chromosome (*N*_*C*_) to those among different chromosomes (*N*_*T*_) for a given bin *i*: ${R}_{i}=\frac{{N}_{T}}{{N}_{C}}$. This ratio represents the *trans-*interaction tendency while it is not confounded by CNV; and (3) copy-number-adjusted *trans-*chromosomal interaction (_adj_nTIF), which is also used to measure a genomic locus’s interaction activity, was calculated as described before^49^.

### Develop ecDNA callers for identifying ecDNA candidates

We derived two ecDNA callers to identify ecDNA candidates; one is based on the logistic regression model, and the other one is based on the convolutional neural networks. Both models predict genome-wide 1 Mb bins that contain ecDNA, as well as cells with presence of ecDNA population-wide. Detailed methodologies for both algorithms are delineated as follows.

#### Logistic regression model-based ecDNA caller

We trained a multivariate logistic regression model using the glm function in R with inferred copy number, hub index and *trans*-to-*cis* contacting bin ratio as predictive variables. The training dataset comprised the well-defined ecMYC in COLO320DM (chr8: 127–128 Mb) and EGFR ecDNA in GBM39 (chr7: 55–56 Mb) as positive data, with the same loci in COLO320HSR and GBM39-ER as negative control. We established a probability threshold for classifying ecDNA presence. Specifically, in COLO320DM/COLO320HSR data, this threshold was determined to be 0.5. In GBM39/GBM39-ER data, the threshold was determined to be 0.95.

#### Deep-learning-model-based ecDNA caller

##### Data preprocessing

To train the deep-learning-based ecDNA caller, we also selected the well-defined ecMYC in COLO320DM (chr8: 127–128 Mb) and EGFR ecDNA in GBM39 (chr7: 55–56 Mb) as positive data, with the same loci in COLO320HSR and GBM39-ER as negative control. We used all autosomes and chromosome *X* to create a Hi-C contact matrix at 1 Mb resolution (3,044 × 3,044) for each cell. We then randomly selected 90% of cells as the training data and kept the remaining 10% of cells as the validation data. For each 1 Mb bin, we retained its local 5 Mb neighborhood region (including the center bin itself), and used both *cis-* and *trans-*contacts (that is, a 5 × 3,044 matrix) as its feature in the neural network model.

##### Model architecture

Our proposed model consists of two sequentially placed convolutional modules that extract features from the binarized Hi-C contact matrices. Each convolutional module consists of a multi-channel convolutional layer (8 channels in the first module, 16 channels in the second module), a batch normalization layer, a rectified linear unit activation function^96^ and a max-pooling^97^ layer sequentially. Convolution kernels scan along the direction of rows in each layer. Large convolutional kernel sizes (5 × 45 in the first module and 1 × 45 in the second layer) are set to improve pattern capture because of sparsity. Max-pooling reduces the size of the matrix to half on its second dimension to keep the most important features and thus improves learning efficiency and propagation speed. Strides and paddings of each layer were designed to balance computational efficiency and information retention and must be compatible with the matrix shapes and max-pooling layers. To enhance robustness and prevent overfitting, a dropout layer^98^ with probability of 0.5 is inserted between convolutional modules.

Subsequently, a two-layer fully connected (dense) network integrates information from multiple sources and makes ternary predictions. The first fully connected layer with a 223 hidden size receives (1) the flattened output of the convolutional modules, (2) the flattened 5 × 5 small contact matrix of the corresponding 5 Mb region (whose diagonal entries denote intra-bin contacts) from binarized 5 × 3,044 Hi-C contact matrices, (3) the hub index (used to measure inequality and heterogeneity of interactions with different chromosomes for certain bins and calculated by Python package ‘pygini’) of the center bin calculated from interaction counts aggregated per chromosome and (4) L1 normalized row means of binarized 5 × 3,044 Hi-C contact matrices. The output passes through a batch normalization layer, a Gaussian error linear unit activation function^99^ and a dropout layer with probability of 0.5. Finally, the second fully connected layer with hidden size 64 produces predictions with a subsequent softmax activation function, in which each prediction contains the three probabilities of ‘none’, ‘ecDNA’ and ‘HSR’ sequentially.

##### Model training and validation

With the package PyTorch^100^, the training process spanned 40 epochs using the mini-batch training strategy (batch size = 32). To ensure robust optimization, we applied the AdamW^101,102^ optimization algorithm with 0.001 weight decay and 0.001 learning rate, and implemented the ‘hard’ bootstrapped cross-entropy loss with parameter *β* equal to 0.99, which is calculated from one-hot-encoded class labels and predictive softmax^103^ probabilities. To efficiently reduce false positives, we compensated the difference in quantities of different labels and in addition forced the model to favor negative prediction (‘none’) using biased class weights, which is incorporated into the loss calculation. Thus, the model receives a greater loss as a penalty when generating any positive prediction (‘ecDNA’ or ‘HSR’). We mainly used confusion matrix and subsequently derived binary and ternary precision, sensitivity, specificity and accuracy as our evaluation metrics, and binary results (‘none’ or ‘ecDNA’ only) from logistic regression as the baseline performance.

##### Prediction of ecDNA

The model scans each Hi-C contact matrix from beginning to end without strides to generate SoftMax probabilities on each 1 Mb bin (except the first two bins of chromosome 1 and the last two bins of chromosome *X* as no padding to the Hi-C matrix is applied) on each single cell. Argmax (the arguments of the maxima) function is used to determine the final predicted class, which is either 0 (‘none’), 1 (‘ecDNA’) or 2 (‘HSR’). Subsequently, results are aggregated to calculate the proportion of cells with ecDNA and/or HSR among the entire cell population.

### Identify significant *trans-*interactions of ecDNA candidate loci

To identify genomic regions that preferentially interact with ecDNA, we first quantify the number of interactions (*N*_*i*_) of 500 kb genomic interval *i* on different chromosomes with ecDNA candidate loci, treating each chromosome separately. The observed interaction frequency (*P*_*i*_) is the proportion of *N*_*i*_ relative to all contacts on the same chromosome: ${P}_{i}=\,\frac{{N}_{i}}{{\sum }_{g=1}^{n}{N}_{g}}$. The expected interaction frequency for interval *i* was calculated as the ratio of the inferred copy number (CN_*i*_) to the total copy number on the same chromosome: ${E}_{i}=\,\frac{{\rm{CN}}_{i}}{{\sum }_{g=1}^{n}{\rm{CN}}_{g}}$, based on the null hypothesis that the interaction frequency for genomic regions with ecDNA is weighted only by its underlying copy number. To identify significant interactions, observed-versus-expected *P* value was calculated based on the binomial distribution model. Multiple testing correction was done by Bonferroni adjustment. Regions with adjusted *P* value < 0.05 were selected as significant interacting regions.

### ecDNA hub analysis

To generate a background distribution for ecDNA hub analysis, we shuffled chromosome identities for all *trans* interactions in each cell and calculated the hub index using R package ineq based on the shuffled contact matrices. *P* value was calculated using the Wilcoxon signed-rank test.

### Analysis of GBM cellular states

To classify single cells within GBM 10x Genomics Multiome or Paired Hi-C RNA datasets into one of four predefined malignant cellular states, we adopted a previously described two-dimensional visualization technique^53^. Briefly, we quantified the gene enrichment score (SC_*j*(*i*)_) for each cell (*i*) against one of the four gene sets (*G*_*j*_) associated with the particular cellular state. This score was calculated as the relative averaged expression (Exp) of *G*_*j*_ in cell *i* compared with a group of genes (*G*_*j*cont_) with a similar level of expression as the control: ${\rm{SC}}_{j(i)}=\frac{{\sum }_{g=1}^{{N}_{j}}{\rm{Exp}}_{g(i)}}{{N}_{j}}-\frac{{\sum }_{g=1}^{{N}_{j}\rm{cont}}{\rm{Exp}}_{g(i)}}{{N}_{{j}\rm{cont}}}$, where *N*_*j*_ and *N*_*j*cont_ are the number of genes in *G*_*j*_ and *G*_*j*cont_. After obtaining scores for all four cellular states, the cells were stratified into OPC/NPC versus AC/MES categories using the differential score $D=\max \left({\rm{SC}}_{\rm{OPC}},\,{\rm{SC}}_{\rm{NPC}}\right)-\max \left({\rm{SC}}_{\rm{AC}},\,{\rm{SC}}_{\rm{MES}}\right)$. For further refinement, OPC/NPC cells were assigned an identity value $C={\log }_{2}(\left|{\rm{SC}}_{\rm{OPC}}-{\rm{SC}}_{\rm{NPC}}\right|+1)$, and AC/MES cells were similarly categorized with $C={\log }_{2}(\left|{\rm{SC}}_{\rm{AC}}-{\rm{SC}}_{\rm{MES}}\right|+1)$. The distribution of cellular states was then plotted in the two-dimensional representation with *D* on the *y*-axis and *C* on the *x*-axis.

For cellular state identification in Droplet Hi-C data, co-embedding with the reference snRNA-seq was performed using the scGAD score. After determining the top 15 nearest snRNA-seq neighbors and their scaled distance-based similarity score (*D*_*m*_) for each Droplet Hi-C cell, the Hi-C gene enrichment score (HSC_*j*(*i*)_) was computed as the sum of the neighbor-weighted enrichment scores: ${\rm{HSC}}_{j\left(i\right)}=\,{\sum }_{g=1}^{15}{\rm{SC}}_{j(g)}x{D}_{m(g)}$.

### Processing and analysis of Paired Hi-C data

Preprocessing and analysis of Hi-C modality in Paired Hi-C are identical as described for Droplet Hi-C. For RNA modality, fastq files were demultiplexed by cellranger-arc, but preprocessed using cellranger (v6.1.2). After obtaining the cell-by-gene matrix, clustering and visualization were performed as described for the 10x Genomics Multiome RNA dataset. As barcodes in the same gel bead for RNA and Hi-C modalities are different, we performed manual pairing to match cell barcodes based on the 10x Genomics Multiome barcodes white list provided in cellranger-arc (10x Genomics).

Integration of the Paired Hi-C RNA dataset with reference datasets was performed by Seurat. First, normalization of gene counts was performed, and the top 2,000 shared variable genes among datasets were selected as integration features. Subsequent canonical correlation analysis allowed projecting all nuclei into a unified embedding space. Anchors (pairs of corresponding cells from distinct datasets) were then discerned through mutual nearest neighbor searching. Anchors with low confidence were excluded, and the shared neighbor between anchors and query cells are computed. Louvain clustering was applied to the shared neighbor graph to discern co-embedded clusters. We calculated overlap coefficients as described in a previous section to compare clustering results from different datasets.

### Analysis of gene expression levels and copy numbers of ecDNA

To calculate the correlation between gene expression level and inferred copy number of genes on ecDNA, we first refined the range of ecDNA at 10 kb resolution. This is based on the observation of the increased intra-ecDNA interaction frequency than interactions with regions on linear genome, irrespective of their genomic distance. Specifically, we enumerated the local interactions within each 10 kb segment of the 1 Mb ecDNA candidates. Subsequently, the contact numbers were smoothed among adjacent bins. By calculating the differential contact numbers between neighboring bins, we determined the changing points of interaction with an average difference cutoff among the entire 1 Mb region. The outermost local maxima and minima were designated as ecDNA boundaries.

With the ecDNA boundaries established, we categorized genes with gene bodies residing within ecDNA as ‘ecDNA genes’. In the case of ecMYC, we further classified genes on GBM39 ecMYC as ‘shared genes’ and genes on non-overlapping regions between GBM39 and GBM39-ER as ‘ecMYC variable genes’, considering the greater ecDNA size in GBM39-ER at the pseudo-bulk level. The SCC between gene expression levels (RPKM) and the average inferred copy number over all 10 kb segments at gene body among all cells was calculated, for a comprehensive representation of the interplay between gene expression and copy number.

### Ethical approval

The GBM specimen collection was approved by the Institutional Review Board (IRB) at the University of Minnesota (IRB number STUDY00012599). The AML and myelodysplastic syndrome specimen collection was approved by the IRB at the University of California, San Diego (IRB number 131550). Before sample collection, a dedicated research coordinator obtained informed consent from each patient, in accordance with the Declaration of Helsinki and appropriate Ethics Committee approval from each partner institution.

### Reporting summary

Further information on research design is available in the Nature Portfolio Reporting Summary linked to this article.

## Online content

Any methods, additional references, Nature Portfolio reporting summaries, source data, extended data, supplementary information, acknowledgements, peer review information; details of author contributions and competing interests; and statements of data and code availability are available at 10.1038/s41587-024-02447-1.

## Supplementary information

Supplementary Information Supplementary Figs. 1–3 and Protocols.

Reporting Summary

Supplementary Table 1 Human cell line mixing metadata.

Supplementary Table 2 Droplet Hi-C mouse cortex metadata.

Supplementary Table 3 ecDNA prediction results.

Supplementary Table 4 Paired Hi-C mouse cortex metadata.

## Source data

Source Data Fig. 1 Statistical source data.

Source Data Fig. 2 Statistical source data.

Source Data Fig. 3 Statistical source data.

Source Data Fig. 4 Statistical source data.

Source Data Fig. 5 Statistical source data.

Source Data Fig. 6 Statistical source data.

Source Data Extended Data Fig. 1 Statistical source data.

Source Data Extended Data Fig. 2 Statistical source data.

Source Data Extended Data Fig. 3 Statistical source data.

Source Data Extended Data Fig. 4 Statistical source data.

Source Data Extended Data Fig. 5 Statistical source data.

Source Data Extended Data Fig. 6 Statistical source data.

Source Data Extended Data Fig. 7 Statistical source data.

Source Data Extended Data Fig. 9 Statistical source data.

Source Data Extended Data Fig. 10 Statistical source data.

## Data Availability

Raw and processed sequencing data generated in this study have been submitted to GEO (accession number GSE253407)^104^. Datasets for bulk in situ Hi-C on cultured cells were downloaded from the 4DN Data Portal with the following accession numbers: K562 (4DNFIIX5BNC9 and 4DNFI244AS29)^105^, GM12878 (4DNFIIS73OJN and 4DNFI3O82QVV)^32^ and HeLa S3 at prometaphase (4DNFIW458FJD)^34^. Other external datasets were downloaded from NCBI GEO with the following accession numbers: in situ Hi-C on WTC-11 (GSE106690)^106^, sci-Hi-C on cell line mixture (GSE84920)^22^, Dip-C on the adult mouse cortex (GSE162511)^17^, HiRES on mouse embryos (GSE223917)^21^, single-cell Hi-C on mouse Th1 cells (GSE48262)^13^, single-nuclei Hi-C on K562 (GSE80006)^16^, Droplet Paired-Tag dataset on the mouse frontal cortex (GSE152020)^40^, 10x Genomics Multiome dataset on the mouse cortex (GSE210749)^107^, 10x Genomics Multiome dataset on COLO320DM and COLO320HSR (GSE160148)^44^ and DOGMA-seq dataset on PBMCs (GSE156478)^57^. The BICCN whole mouse brain sn-m3C-seq datasets^37^ and BICCN MOp 10x snRNA-seq data^108^ were downloaded via the NeMO archive (https://assets.nemoarchive.org/dat-sig83t9; https://assets.nemoarchive.org/dat-ch1nqb7). The 10x Genomics Multiome dataset on PBMCs was downloaded from the 10x Genomics dataset portal (https://www.10xgenomics.com/en/datasets). The human reference genome (GRCh38/hg38, https://hgdownload.soe.ucsc.edu/goldenPath/hg38/bigZips)^109^ and the mouse reference genome (GRCm38/mm10, https://hgdownload.soe.ucsc.edu/goldenPath/mm10/bigZips)^110^ were from UCSC. Source data are provided with this paper.

## Code availability

Scripts and code are available via GitHub at https://github.com/Xieeeee/Droplet-Hi-C (ref. ^111^). The ecDNA callers are available via GitHub at https://github.com/HuMingLab/ecDNAcaller (ref. ^112^). Scripts to analyze the multi-way interaction hub are available via GitHub at https://github.com/HuMingLab/Multiway.hub (ref. ^113^).
