# Author Correction: Droplet Hi-C enables scalable, single-cell profiling of chromatin architecture in heterogeneous tissues

**DOI:** 10.1038/s41587-025-02697-7

**Published:** 2025-05-12

**Authors:** Lei Chang, Yang Xie, Brett Taylor, Zhaoning Wang, Jiachen Sun, Ethan J. Armand, Shreya Mishra, Jie Xu, Melodi Tastemel, Audrey Lie, Zane A. Gibbs, Hannah S. Indralingam, Tuyet M. Tan, Rafael Bejar, Clark C. Chen, Frank B. Furnari, Ming Hu, Bing Ren

**Affiliations:** 1 Department of Cellular and Molecular Medicine, University of California, San Diego, La Jolla, CA USA; 2 Biomedical Sciences Graduate Program, University of California, San Diego, La Jolla, CA USA; 3 Medical Scientist Training Program, University of California, San Diego, La Jolla, CA USA; 4 Department of Quantitative Health Sciences, Lerner Research Institute, Cleveland Clinic Foundation, Cleveland, OH USA; 5 Systems Biology and Bioinformatics PhD Program, Case Western Reserve University School of Medicine, Cleveland, OH USA; 6 Bioinformatics and Systems Biology Program, University of California, San Diego, La Jolla, CA USA; 7 Moores Cancer Center, UC San Diego, La Jolla, CA USA; 8 Department of Neurosurgery, University of Minnesota, Minneapolis, MN USA; 9 Department of Medicine, University of California, San Diego School of Medicine, La Jolla, CA USA; 10 Center for Epigenomics, Institute for Genomic Medicine, School of Medicine, University of California, San Diego, La Jolla, CA USA

**Keywords:** Next-generation sequencing, Predictive markers

Correction to: *Nature Biotechnology* 10.1038/s41587-024-02447-1, published online 18 October 2024.

In the version of the article initially published, in the Methods section, both mentions of “MboII” should have read “MboI (NEB, R0147M)” and have now been corrected in the HTML and PDF versions of the article.

