# Supplementary material for: Droplet Hi-C enables scalable, single-cell profiling of chromatin architecture in heterogeneous tissues
Source: Nat Biotechnol. 2024 Oct 18;43(10):1694–707. doi: 10.1038/s41587-024-02447-1 (PMC12520981; doi:10.1038/s41587-024-02447-1)
Supplement: Supplementary file 1 — Supplementary Figs. 1–3 and Protocols. [file 41587_2024_2447_MOESM1_ESM.pdf]

nature biotechnology

Supplementary information

https://doi.org/10.1038/s41587-024-02447-1

Droplet Hi-C enables scalable, single-cell profiling of chromatin architecture in heterogeneous tissues

In the format provided by the authors and unedited

Supplementary Information

Supplementary Figures

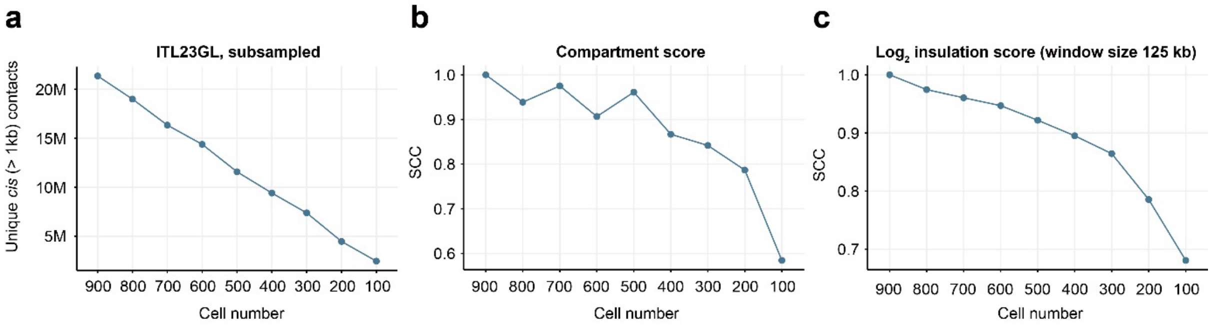

Supplementary Fig. 1 Subsampling of the Droplet Hi-C data shows that chromatin compartments and TADs can be robustly mapped from as few as 400 cells.

a, Scatter plot showing the relationship between number of *cis*-long contacts and subsampled cell number in ITL23GL cell type in adult mouse cortex Droplet Hi-C data. **b,** Scatter plot showing the Spearman's correlation coefficients (SCC) of compartment score from subsampled pseudo-bulk Droplet Hi-C profiles with pseudo-bulk profiles from all cells. **c,** Scatter plot showing the Spearman's correlation coefficients (SCC) of insulation score from subsampled pseudo-bulk profiles with the profile from all cells.

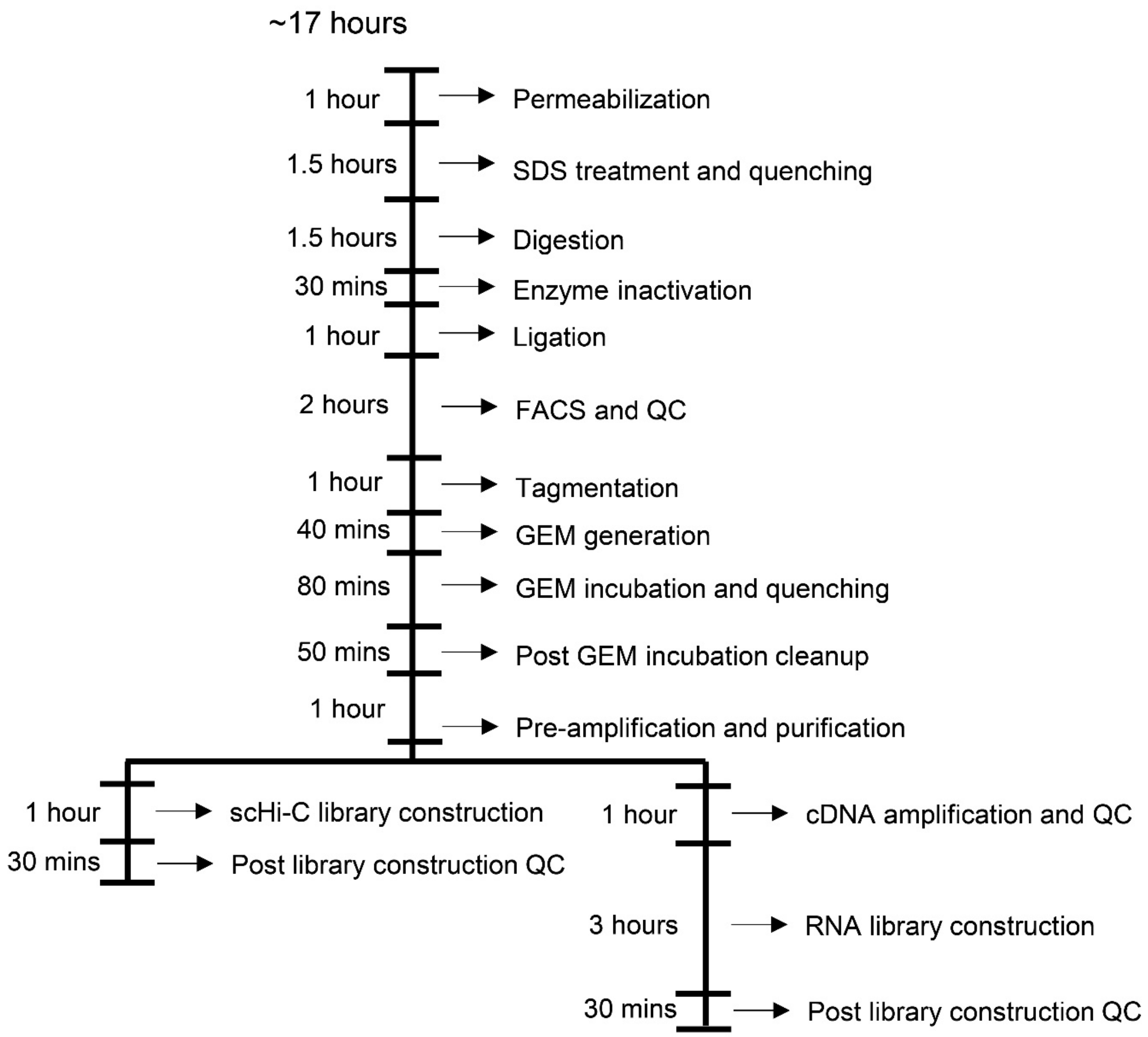

Supplementary Fig. 2 Workflow of Paired Hi-C.

Detailed workflow describing the end-to-end procedure of Paired Hi-C and time cost for each step.

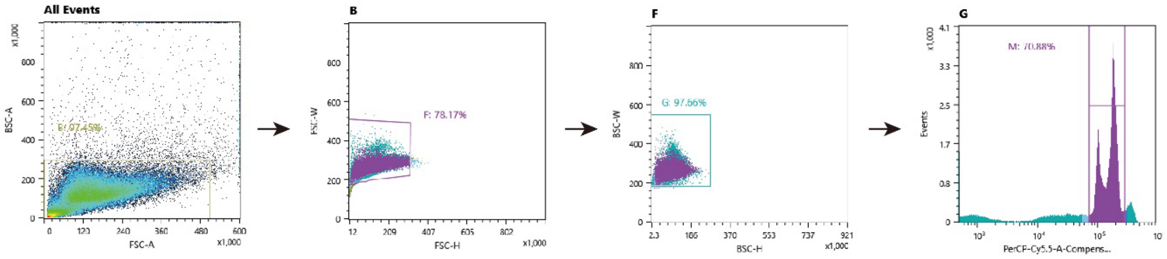

Supplementary Fig. 3 Nuclei gating strategy for 7'AAD stained nuclei for HeLa S3 and mESC mixing experiment.

After Hi-C ligation, nuclei were stained with 7'AAD and proceed to fluorescence-activated nuclei sorting. Potential nuclei were retained based on forward scatter (FSC) area and back scatter (BSC) area (left-most scatter plot). Next, potential debris and doublets were filtered based on BSC and FSC signal width and height (two middle scatter plots). Finally, diploid (mESC) and hypertriploid (HeLa S3) nuclei were sorted into collection tube (right-most scatter plot) according to fluorescence from 7'AAD.

Supplementary Protocols

Cell culture

HeLa S3 (human, ATCC CCL-2.2) cells were cultured according to standard procedures in high glucose DMEM (Sigma-Aldrich, D6429) supplemented with 10% fetal bovine serum (FBS; Omega Scientific, FB-02) and 1% Penicillin-Steptomycin-Glutamine (ThermoFisher Scientific, 10378016) at 37°C with 5% CO_2.

mESCs were maintained in feeder-free and serum-free 2i medium at 37 °C with 5% CO_2. To isolate nuclei, mESCs were dissociated with Accutase (Innovative Cell Technologies, AT104), collected by centrifugation.

K562 cells were cultured at 37°C, 5% CO_2 in RPMI-1640 (ATCC, 30-2001) supplemented with 1% Penicillin-Steptomycin-Glutamine and 10% FBS.

GM12878 cells were cultured at 37°C, 5% CO_2 in RPMI-1640 supplemented with 1% Penicillin-Steptomycin-Glutamine and 15% FBS.

WTC-11 iPSCs (a gift from the Yin Shen lab at UCSF) containing an inducible NGN2 cassette inserted into the AAVS1 safe harbor locus were maintained in Essential 8 Flex medium (Thermo Fisher, A2858501) on culture dishes coated with Matrigel (Corning, 354277). Cells were cultured with 10 uM ROCK inhibitor (STEMCELL Technologies, Y-27632) for 24h after seeding, and fed daily with fresh E8 Flex medium

COLO320DM (ATCC, CCL-220) and COLO320HSR (ATCC, CCL-220.1) were maintained in RPMI-1640 supplemented with 10% FBS (ThermoFisher, S11550H) and 1% penicillin-streptomycin (ThermoFisher, 15140122). Media was refreshed every 3-4 days, and cells were passaged once per week using Accutase (VWR, AT104) for dissociation.

Patient-derived xenograft (PDX) model GBM39 (Mayo Clinic Hospital) was cultured in DMEM/F12 (ThermoFisher, 11320033), 20ng/mL EGF (STEMCELL Technologies, 78006), 20ng/ml FGF (STEMCELL Technologies, 78134.1), 10% B27 Supplement without Vitamin A (ThermoFisher, 12587010), and 1% penicillin-streptomycin solution at 37°C with 5% CO_2. GBM39-ERL was under continuous treatment with 5μM erlotinib (LC Laboratories, E-4007). GBM39 cell lines were cultured as neurospheres.

Adherent cells were washed once with 1x PBS, trypsinized with 0.25% trypsin–EDTA (ThermoFisher Scientific, 25200056), spun down at 500 x g for 5 min, resuspended in culture medium, and spun down again at 500 x g for 5 min. Suspension cells were spun down at 500x g for 5 min to be collected from the culture medium. The cell pellets were resuspended in 1 million cells/mL 1x PBS (pH=7.4) for crosslinking.

Nuclei preparation from the mouse cortex

All animal work described in this manuscript has been approved and conducted under the oversight of the Institutional Animal Care and Use Committee at the University of California, San Diego. Male C57BL/6J mice were purchased from the Jackson Laboratory (000664) at 7 weeks of age and were housed in the animal facility at University of California, San Diego, under a 12-h light/12-h dark cycle in a temperature-controlled room with *ad libitum* access to water and food until euthanasia and tissue collection at 8 weeks of age. The isocortex was dissected from 8-week-old male mice, snap-frozen in liquid nitrogen and stored at −80 °C before proceeding to nuclei extraction.

Single-nuclei suspensions were prepared from fresh tissues by dounce homogenization in douncing buffer (0.25 M sucrose (Sigma, S7903), 25 mM KCl (Invitrogen, AM9640G), 5 mM $MgCl_2$ (Invitrogen, AM9530G), 10 mM Tris-HCl (pH 7.5) (ThermoFisher Scientific, 15567027), 1 mM DTT (Sigma, D9779), 1x protease inhibitor (Roche, 5056489001), 0.5 U/μL

RNaseOUT(Invitrogen, 10777019), 0.5 U/µL SUPERaseIn inhibitor (Invitrogen, AM2694) and 0.1% Triton-X100 (Sigma, 93443)). The nuclei suspension was then filtered through a 30-µm Cell-Tric filter (Sysmex) and centrifuged for 10 min at 300 x g at 4°C. Cell pellets was washed once with douncing buffer without Triton-X100, centrifuged again and resuspended in 1 million cells/mL 1x PBS (pH=7.4) for crosslinking.

Metaphase spread cell preparation

One million cells were seeded into a single well of a 6-well plate coated with Matrigel (Corning, 354277). Cells were incubated in 100 ng/mL of Karyomax (Gibco, 15212-012) in standard growth media for 10 hours. Cells were washed with PBS, lifted with Accutase (Innovative Cell Technologies, AT-104), and collected into a 15 ml conical tube. Cells were spun down in the centrifuge at 1000 rpm for 5 minutes. The supernatant was aspirated, and the cell pellet was dislodged by gently flicking the conical tube. Cells were incubated in 1 mL of warm 0.075 M KCl. 200 uL of cold Carnoy's Fixative (3:1 mixture of methanol and acetic acid) was added to the cell mixture, which was mixed by gentle inversion. Cells were spun down at 1000 rpm for 5 minutes, the supernatant was aspirated, and the pellet was resuspended in 1 mL of cold Carnoy's Fixative. The cell mixture was transferred to a 1.5 mL Eppendorf tube and re-spun at 1000 rpm for 5 minutes, and the supernatant was aspirated. Fixed mitotic cells were resuspended in 40 uL of Carnoy's fixative and were either used immediately for metaphase spreads or stored at -20C. For each metaphase spread, 4 uL of cells were added to glass coverslips in a dropwise manner.

Fluorescence *in situ* hybridization (FISH)

Slides containing fixed cells in interphase and metaphase or 16-µm tissue slices were briefly equilibrated in 2X SSC, followed by dehydration in 70%, 85%, and 100% ethanol for 2 minutes each. FISH probes in hybridization buffer (Empire Genomics) were then added to the slide, which was covered with a coverslip. The sample was denatured on a hotplate at 75°C for 3 minutes for fixed cell samples and 7 minutes for tissue samples, followed by hybridization at

37°C overnight. After removing the coverslip, the sample was washed once with 0.4X SSC containing 0.3% IGEPAL and once with 2X SSC containing 0.1% IGEPAL, each for 2 minutes. Finally, the sample was mounted using ProLong Diamond Antifade Mountant with DAPI (Invitrogen, P36966) before imaging. Images were acquired using a Zeiss LSM880 Airyscan confocal microscope with a 63x Plan-APOChromat NA 1.4 oil lens. Representative images were selected from the Z-stack with the best brightness.

The UCSD Cytogenetics Laboratory performed FISH experiments on GBM39 and GBM39-ER cells using a *MYC* dual color break-apart probe set. This probe set detects gene rearrangement at the *MYC* locus on chromosome 8 at band q24 (MYC: Abbott Molecular, Inc.).

Bulk *in situ* Hi-C of HeLa S3 cells

<u>Replicate 1</u>

Hi-C libraries were generated as described before with minor modifications[1]. Briefly, 1 million crosslinked HeLa S3 cells were lysed with 300 μl ice cold lysis buffer (10 mM Tris-HCl (pH 8.0), 10 mM NaCl, 0.2% IPEGAL CA-630 with 1x protease inhibitors) on ice for 15 minutes, washed once with 500μl lysis buffer and pelleted at 4°C 2,500g for 5 min. Cell pellets were resuspended in 50μl 0.5% SDS and incubated for 10 min at 62 °C followed by quenching with 160 μl 1.56% Triton X-100 for 15 min at 37 °C. The chromatin was digested overnight at 37 °C with shaking (1,000 r.p.m.) with 100 U MboI in 25 μl 10x NEBuffer 2. Digested fragments were filled in with biotin-14-dATP at 37 °C 900 RPM for 2 hours and subsequently ligated with T4 DNA ligase (NEB) for 4 h at 23 °C with 300 r.p.m. gentle rotation. After the ligation step, nuclei were digested by 50 μl proteinase K (20 mg/ml) for 30 min at 55 °C. The DNA was purified by ethanol precipitation and resuspended in 130 μl 10 mM Tris-HCl (pH 8.0) and sonicated by a Covaris M220 sonicator with the parameters: duty cycle, 10%; power, 50; cycles per burst, 200; time, 70 s. Biotin-labeled free DNA ligation junctions were enriched by 150 μl MyOne T1 streptavidin Dynabeads. Once bound to the streptavidin beads, the biotin-labeled DNA was

subjected to end repair & biotin removal from free ends, A-tailing followed by ligation of Illumina indexed TruSeq adapters. The DNA is then released from the beads by a 10-minute incubation at 98 °C. Smaller DNA fragments were removed by a 0.8x SPRI bead purification. The DNA is then PCR amplified. Final libraries were purified by SPRI bead based dual size selection (0.55:1 and 0.75:1), quantified and submitted for pair-end sequencing.

Replicate 2

Bulk Hi-C was performed as previously described[2] with minor modification. Briefly, crosslinked HeLa S3 cells nuclei were extracted with10mM Tris-HCl pH8.0, 10mM NaCl, and 0.2% Igepal CA630 on ice. The chromatin in nuclei were sheared with two restriction enzymes, MboI (NEB, R0147) and HinfI (NEB, R0155L). Chromatin was end repaired with Large (Klenow) Fragment (NEB, M0210) with Biotin incorporated (Jena Bioscience JBS-NU-835-BIO14-L), and further ligated with T4 DNA ligase (NEB, M0202). Chromatin was decrosslinked in proteinase K solution (QIAGEN 19133), with DNA released and further purified. Sonication was performed in a Covaris ultrasonicator to shear DNA into an average size of 300-500bp. Bead-based (Beckman B23319) size selection was performed to remove out-of-range DNA fragments. Biotin-labeled DNA was pulled down with Dynabeads MyOne Streptavidin T1 beads (Life technologies, 65602). Sequencing library was prepared for the Biotin-enriched DNA using KAPA HyperPrep kit (KK8502) and xGen™ UDI-UMI Adapters (IDT 10006914) following the user manuals. The library was sequenced on Illumina platform.

Analysis of 10x Genomics Multiome datasets

10x Genomics Multiome fastq files for the GBM patient sample were demultiplexed and pre-processed with cellranger-arc (v2.0.0). For human PBMC 10x Genomics Multiome data, the processed and filtered matrices was downloaded directly from 10x data portal. Clustering of single-nucleus transcriptomic or chromatin accessibility data were performed in R using Seurat (v4.1.0) or Signac (v1.6.0)[3, 4]. For transcriptomic data, gene counts were normalized and scaled, and the top 2,500 variable genes were selected for dimension reduction by PCA. The

first 30 principal components were used for UMAP visualization and Louvain clustering. Potential doublets were identified and removed using Scrublet[5]. For chromatin accessibility data, cell-by-peak matrices were normalized by the two-step term frequency-inverse document frequency (TF-IDF). The top 95% of genomic bins were selected for linear dimension reduction, again followed by UMAP visualization and Louvain clustering. Gene activity scores were computed by ATAC signal density in promoter and gene body regions.

Gene Ontology (GO) enrichment

We perform Gene Ontology enrichment analysis for DE genes overlap with switched compartments in GBM cellular states using Enrichr in R[6]. The gene set 'GO_Biological_Process_2018' was used with default parameters. Combined score is defined as the Fisher's exact test *P* value multiplied with the Z-score of the deviation from the expected rank. The results are used to generate plot in Extended Data Fig 7k.

Single-cell genotyping by Droplet Hi-C data

Frequently observed mutations in AML were sequenced using Hematologic Malignancy Comprehensive Panel for the patient BMMC sample. To detect malignant cells carrying the detected mutations from Droplet Hi-C data, cellular barcode information was appended to the mapped bam files as an extra tag. To ensure comparability between pre- and post-treated samples, bam files were subsampled to a uniform sequencing depth. we employed a previously reported custom script to count reads for both the wild-type and mutant alleles, considering only barcodes that met predefined quality standards[7]. For each cell and mutational site, we then summarized the detected mutant and wild-type reads. Given the limited sequencing coverage, we defined potential malignant cells as cell carrying at least one mutant read.

References

1. Rao, S.S. et al. A 3D map of the human genome at kilobase resolution reveals principles of chromatin looping. *Cell* **159**, 1665-1680 (2014).
2. Xu, J. et al. Subtype-specific 3D genome alteration in acute myeloid leukaemia. *Nature* **611**, 387-398 (2022).
3. Cao, Y. et al. Integrated analysis of multimodal single-cell data with structural similarity. *Nucleic Acids Research* **50**, e121-e121 (2022).
4. Stuart, T., Srivastava, A., Madad, S., Lareau, C.A. & Satija, R. Single-cell chromatin state analysis with Signac. *Nature Methods* **18**, 1333-1341 (2021).
5. Wolock, S.L., Lopez, R. & Klein, A.M. Scrublet: computational identification of cell doublets in single-cell transcriptomic data. *Cell systems* **8**, 281-291. e289 (2019).
6. Kuleshov, M.V. et al. Enrichr: a comprehensive gene set enrichment analysis web server 2016 update. *Nucleic Acids Research* **44**, W90-W97 (2016).
7. van Galen, P. et al. Single-Cell RNA-Seq Reveals AML Hierarchies Relevant to Disease Progression and Immunity. *Cell* **176**, 1265-1281.e1224 (2019).
