# Supplementary material for: Droplet Hi-C enables scalable, single-cell profiling of chromatin architecture in heterogeneous tissues
Source: Nat Biotechnol. 2024 Oct 18;43(10):1694–707. doi: 10.1038/s41587-024-02447-1 (PMC12520981; doi:10.1038/s41587-024-02447-1)
Supplement: Supplementary file 2 — Reporting Summary [file 41587_2024_2447_MOESM2_ESM.pdf]

nature portfolio

Corresponding author(s): Bing Ren

Last updated by author(s): Sep 14, 2024

Reporting Summary

Nature Portfolio wishes to improve the reproducibility of the work that we publish. This form provides structure for consistency and transparency in reporting. For further information on Nature Portfolio policies, see our Editorial Policies and the Editorial Policy Checklist.

Statistics

For all statistical analyses, confirm that the following items are present in the figure legend, table legend, main text, or Methods section.

n/a	Confirmed	
☐	☒	The exact sample size (*n*) for each experimental group/condition, given as a discrete number and unit of measurement
☐	☒	A statement on whether measurements were taken from distinct samples or whether the same sample was measured repeatedly
☐	☒	The statistical test(s) used AND whether they are one- or two-sided<br>*Only common tests should be described solely by name; describe more complex techniques in the Methods section.*
☐	☒	A description of all covariates tested
☐	☒	A description of any assumptions or corrections, such as tests of normality and adjustment for multiple comparisons
☐	☒	A full description of the statistical parameters including central tendency (e.g. means) or other basic estimates (e.g. regression coefficient) AND variation (e.g. standard deviation) or associated estimates of uncertainty (e.g. confidence intervals)
☐	☒	For null hypothesis testing, the test statistic (e.g. *F*, *t*, *r*) with confidence intervals, effect sizes, degrees of freedom and *P* value noted<br>*Give P values as exact values whenever suitable.*
☒	☐	For Bayesian analysis, information on the choice of priors and Markov chain Monte Carlo settings
☒	☐	For hierarchical and complex designs, identification of the appropriate level for tests and full reporting of outcomes
☐	☒	Estimates of effect sizes (e.g. Cohen's *d*, Pearson's *r*), indicating how they were calculated

Our web collection on statistics for biologists contains articles on many of the points above.

Software and code

Policy information about availability of computer code

Data collection	Illumina bcl2fastq2 (v2.19.0.316)
Data analysis	10XGenomics cellranger (v6.1.2), 10XGenomics cellranger-atac (v2.0.0), 10XGenomics cellranger-arc (v2.0.0), Python (3.11.8), R (4.2.3), Bowtie (v1.3.0), BWA-MEM (v0.7.17), Trim-Garole (v0.6.10), Samtools (v1.14), deepTools (v3.5.3), IGV (v.2.15.4), NeoLoopFinder (v0.4.3), EagleC (v0.1.9), Seurat (v4.1.0), Signac (v1.6.0), Scanpy (v1.7.2), harmonypy (v0.0.9), PyNNDescent (v0.5.6), cooltools (v0.5.1), Cooler (v0.8.10), Pairtools (v0.3.0), bedtools (v2.27.1), Matplotlib (v3.5.1), igraph (v0.9.9), leidenalg (v0.8.8), scHiCluster (v1.3.4), TopDom (v0.0.2), rGREAT (v1.26.0), enrichR (v3.2), ineq (v0.2-13), pygini (v1.0.1), PyTorch, Sony SH800 Cell Sorter Software (v2.1.6).<br>Custom scripts and code to reproduce figures are available at: https://github.com/Xieeeee/Droplet-Hi-C. The ecDNA callers are available at https://github.com/HuMingLab/ecDNAcaller. Scripts to analyze multi-way interactions hub is available at: https://github.com/HuMingLab/Multiway.hub

For manuscripts utilizing custom algorithms or software that are central to the research but not yet described in published literature, software must be made available to editors and reviewers. We strongly encourage code deposition in a community repository (e.g. GitHub). See the Nature Portfolio guidelines for submitting code & software for further information.

Data

Policy information about availability of data

All manuscripts must include a data availability statement. This statement should provide the following information, where applicable:

- Accession codes, unique identifiers, or web links for publicly available datasets
- A description of any restrictions on data availability
- For clinical datasets or third party data, please ensure that the statement adheres to our policy

Raw and processed sequencing data generated in this study have been submitted to GEO (accession number GSE253407). Datasets for bulk in situ Hi-C on cultured cells were downloaded from the 4DN data portal with the following accession number: K562 (4DNFIIX5BNC9 and 4DNFI244AS29), GM12878 (4DNFIIS73OJN and 4DNFI3O82QVV) and HeLa S3 at prometaphase (4DNFIW458FJD). Other external datasets were downloaded from NCBI GEO with the following accession numbers: in situ Hi-C on WTC-11 (GSE106690), sci-Hi-C on cell lines mixture (GSE84920), Dip-C on adult mouse cortex (GSE162511), HiRes on mouse embryos (GSE223917), single cell Hi-C on mouse Th1 cells (GSE48262), single-nuclei Hi-C on K562 (GSE80006), Droplet Paired-Tag dataset on mouse frontal cortex (GSE152020), 10x Genomics Multiome dataset on mouse cortex (GSE210749), 10x Genomics Multiome dataset on COLO320DM and COLO320HSR (GSE160148), DOGMA-seq dataset on PBMCs (GSE156478). The BICCN whole mouse brain sn-m3C-seq datasets and BICCN MOp 10x snRNA-seq data were downloaded via the NeMO archive (https://assets.nemoarchive.org/dat-sig83t9; https://assets.nemoarchive.org/dat-ch1nqb7). 10x Genomics Multiome dataset on PBMCs in downloaded from the 10x Genomics dataset portal (https://www.10xgenomics.com/en/datasets). Human reference genome (GRCh38/hg38, https://hgdownload.soe.ucsc.edu/goldenPath/hg38/bigZips) and mouse reference genome (GRCm38/mm10, https://hgdownload.soe.ucsc.edu/goldenPath/mm10/bigZips) are from UCSC. Source data are provided with this paper.

Research involving human participants, their data, or biological material

Policy information about studies with human participants or human data. See also policy information about sex, gender (identity/presentation), and sexual orientation and race, ethnicity and racism.

Reporting on sex and gender	Patients where the samples are collected from were de-identified.
Reporting on race, ethnicity, or other socially relevant groupings	Race, ethnicity, and other social groupings were not considered in and did not impact our study design.
Population characteristics	Population characteristics were not considered in and did not impact our study design.
Recruitment	For GBM sample, participates are recruited as patients with GBM for surgery. Each patient was consented by a dedicated research coordinator prior to collection. There was no self-selection bias or other bias that was likely to impact the results. For AML / MDS sample, the patient that provided the samples used in this study was asked to participate in the tissue bank for UCSD patients with hematologic disorders as all patient undergoing procedures in our clinic are. The samples from this patient were selected for study because of the presence of double minute chromosomes (ecDNA) noted on routine clinical testing. There was no self-selection bias or other bias that was likely to impact the results.
Ethics oversight	The GBM specimen collection was approved by the Institutional Review Board (IRB) at the University of Minnesota. The AML and MDS specimen collection was approved by the IRB at the University of California, San Diego.

Note that full information on the approval of the study protocol must also be provided in the manuscript.

Field-specific reporting

Please select the one below that is the best fit for your research. If you are not sure, read the appropriate sections before making your selection.

☒ Life sciences ☐ Behavioural & social sciences ☐ Ecological, evolutionary & environmental sciences

For a reference copy of the document with all sections, see nature.com/documents/nr-reporting-summary-flat.pdf

Life sciences study design

All studies must disclose on these points even when the disclosure is negative.

Sample size	Sample size was determined based on prior published data from similar experiments (Liu et.al., Science, 2023). To evaluate robustness of the methods, each set of experiment was carried out with tissues samples dissected from at least two individuals. For patient samples, due to the difficulties in obtaining samples, samples from single patient are used in each experiment.
Data exclusions	For Droplet Hi-C experiment, low quality single nuclei (low number of contacts) were excluded from downstream analysis as outlined in the Methods section. For 10xMultiome or Paired Hi-C experiments, nuclei with inadequate number of genes detected were excluded from downstream analysis as outlined in the Methods section.
Replication	At least two biological replicates were performed for each set of experiment except for patient samples. For each patient sample, only one biological replicate is collected but at least two technical replicates were performed. All datasets from independent replicates showed similar results.

Randomization	Allocation was random.
Blinding	The experiments were not blinded since identities of tissue regions are necessary to evaluate the specificity and sensitivity of the method. Clustering of single-nuclei transcriptome and chromatin structure data were unsupervised.

Reporting for specific materials, systems and methods

We require information from authors about some types of materials, experimental systems and methods used in many studies. Here, indicate whether each material, system or method listed is relevant to your study. If you are not sure if a list item applies to your research, read the appropriate section before selecting a response.

Materials & experimental systems

n/a	Involved in the study	
☒	☐	Antibodies
☐	☒	Eukaryotic cell lines
☒	☐	Palaeontology and archaeology
☐	☒	Animals and other organisms
☒	☐	Clinical data
☒	☐	Dual use research of concern
☒	☐	Plants

Methods

n/a	Involved in the study	
☒	☐	ChIP-seq
☐	☒	Flow cytometry
☒	☐	MRI-based neuroimaging

Eukaryotic cell lines

Policy information about cell lines and Sex and Gender in Research

Cell line source(s)	Mouse embryonic stem cells (mESC) used in this study is a hybrid F123 mESC line (F1 Mus musculus castaneus×S129/SvJae, maternal 129/Sv, paternal CAST) and is from Rudolf Jaenisch's laboratory at the Whitehead Institute at MIT. WTC11 iPSCs containing an inducible NGN2 cassette inserted into the AAVS1 safe harbor locus is from Yin Shen lab, UCSF. HeLa S3, K562, GM12878, COLO320DM and COLO320HSR are from ATCC. Patient-derived xenograft (PDX) model GBM39 is from Mayo Clinic Hospital.
Authentication	Cells were not authenticated.
Mycoplasma contamination	Cells were not tested for mycoplasma.
Commonly misidentified lines (See ICLAC register)	None of the cell lines used are listed in the ICLAC database.

Animals and other research organisms

Policy information about studies involving animals; ARRIVE guidelines recommended for reporting animal research, and Sex and Gender in Research

Laboratory animals	All animal work described in this manuscript has been approved and conducted under the oversight of the Institutional Animal Care and Use Committee at the University of California, San Diego. Male C57BL/6J mice were purchased from the Jackson Laboratory (000664) at 7 weeks of age and were housed in the animal facility at University of California, San Diego, under a 12-h light/12-h dark cycle in a temperature-controlled room with controlled temperature (20–22 °C) and humidity (30–70%), and ad libitum access to water and food until euthanasia and tissue collection at 8 weeks of age. The isocortex was dissected from 8-week-old male mice, snap-frozen in liquid nitrogen and stored at −80 °C before proceeding to nuclei extraction.
Wild animals	The study did not involve wild animals.
Reporting on sex	All replicates used in this study are male C57BL/6J mice
Field-collected samples	The study did not involve field collected samples
Ethics oversight	All animal work described in this manuscript has been approved and conducted under the oversight of the UC San Diego Institutional Animal Care and Use Committee.

Note that full information on the approval of the study protocol must also be provided in the manuscript.

Plants

Seed stocks: NA

Novel plant genotypes: NA

Authentication: NA

Flow Cytometry

Plots

Confirm that:

☒ The axis labels state the marker and fluorochrome used (e.g. CD4-FITC).

☒ The axis scales are clearly visible. Include numbers along axes only for bottom left plot of group (a 'group' is an analysis of identical markers).

☒ All plots are contour plots with outliers or pseudocolor plots.

☒ A numerical value for number of cells or percentage (with statistics) is provided.

Methodology

Sample preparation: Single-nuclei suspensions were prepared from fresh tissues by dounce homogenization in douncing buffer (0.25 M sucrose (Sigma, S7903), 25 mM KCl (Invitrogen, AM9640G), 5 mM MgCl2 (Invitrogen, AM9530G), 10 mM Tris-HCl (pH 7.5) (ThermoFisher Scientific, 15567027), 1 mM DTT (Sigma, D9779), 1× protease inhibitor (Roche, 5056489001), 0.5 U/μL RNaseOUT(Invitrogen, 10777019), 0.5 U/μL SUPERaseIn inhibitor (Invitrogen, AM2694) and 0.1% Triton-X100 (Sigma, 93443)). The nuclei suspension was then filtered through a 30-μm Cell-Tric filter (Sysmex) and centrifuged for 10 min at 300 x g at 4°C. Cell pellets was washed once with douncing buffer without Triton-X100, centrifuged again and resuspended in 1 million cells/mL 1× PBS (pH=7.4) for crosslinking.
Cells were crosslinked in 1% formaldehyde, which was diluted from 37% formaldehyde with 1× PBS (pH=7.4), and incubated at room temperature for 10 min. After crosslinking, the reactions were quenched in 200 mM glycine and incubated at room temperature for 5 min. Quenched reactions were spun down at 1,000 × g for 5 min at 4°C, resuspended using 1% BSA in 1× PBS (pH=7.4) to wash twice. One million cells were aliquoted into each tube. The cells were spun once again at 1,000 × g for 5 min, supernatant was removed, and the pellet was flash frozen in liquid nitrogen, and finally stored indefinitely at -80°C.
Cell pellets were lysed with pre-cold 300 μL lysis buffer (10 mM Tris-HCl, pH 8.0 (ThermoFisher Scientific, 15568025), 10 mM NaCl (Sigma, S5150), 0.2% Igepal CA630 (Sigma, I8896), 1× protease inhibitor (Roche, 5056489001)) on ice for 45 min, then centrifuged at 1,000 × g for 5 min at 4°C to collect nuclei, and washed once with 200 μL lysis buffer. The nuclei were resuspended with 50 μL 0.5% SDS and incubated at 62°C for 10 min on Thermomixer. Then, we added 145 μL of nuclease-free H2O and 25 μL 10% Triton X-100 (Sigma, 93443) to quench SDS and incubated samples at 37°C for 15 min on Thermomixer at 300 rpm. The samples were added 27 μL of 10× CutSmart Buffer and three restriction enzymes, including 50 U DpnII (NEB, R0543L), 62.5U MboII and 7.5U NlaIII (NEB, R0125L), and then incubated at 37°C for 90 min on Thermomixer at 550 rpm. The enzymes were deactivated at 65°C for 20 min and then cooled down to room temperature. The nuclei were collected by centrifugation at 1000 × g for 5 min at 4°C, washed once with 200 μL ligation buffer (100 μL 10× T4 DNA ligase buffer (NEB, B0202S), 5 μL 20 mg/mL bovine serum albumin (BSA; NEB, B9000S), 865 μL H2O) and ligation reaction was performed with 200 μL ligation buffer and 20 μL T4 DNA ligase (NEB, M0202L) at 37°C for 40 min on Thermomixer at 300 rpm.
The ligated nuclei pellets were resuspended in 1 mL 1% BSA in PBS (diluted from 10% BSA in PBS (Sigma, A1595) using 1× PBS (pH=7.4)) each tube, added 1 μL 1,000× 7-AAD (Invitrogen, A1310) and sorted by fluorescence-activated nuclei sorting with an SH800 cell sorter (Sony) for the isolation of single nuclei.

Instrument: SH800 Cell Sorter (Sony)

Software: Data analysis and display are performed in the SH800 software.

Cell population abundance: We used 7-AAD to stain intact nuclei and separated them from debris, without separating identities of different cell types. Using 'purity' mode in sorting nuclei, we collected 200,000-300,000 nuclei for each sample for down-streaming processing.

Gating strategy: First, potential nuclei were identified using forward scatter (FSC) area and back scatter (BSC) area. Next, potential doublets were removed based on BSC and FSC signal width. Finally, diploid nuclei (2n) were sorted into each tube for down-streaming processing.

☒ Tick this box to confirm that a figure exemplifying the gating strategy is provided in the Supplementary Information.
